# The fast and the focused: Balancing timely and accurate classification of deforestation and degradation drivers using remote sensing

**DOI:** 10.1371/journal.pone.0340610

**Published:** 2026-02-06

**Authors:** Amandine Debus, Emilie Beauchamp, Emily R. Lines

**Affiliations:** 1 Department of Geography, University of Cambridge, Cambridge, United Kingdom; 2 International Institute for Sustainable Development, Geneva, Switzerland; University of Santiago, CHILE

## Abstract

Identifying drivers of deforestation is crucial for developing targeted conservation and land management strategies, and satellite data provide a long time series of data to understand deforestation dynamics. However, the timing of imagery after forest loss may affect classification accuracy, and optimal timing may be different for different drivers. Studies of broad-scale drivers across large and pan-tropical regions have shown that using time series can improve driver classification from satellite imagery, but requiring multi-year information means waiting longer after forest loss to classify what drives it. Our previously introduced model, Cam-ForestNet, was developed to use single-date imagery to classify fifteen direct detailed deforestation and degradation drivers for Cameroon. Here, we test whether the overall and per-class classification performance of Cam-ForestNet can be improved by either using imagery taken longer after a forest loss event or by incorporating a greater number of images, with performance evaluated using macro-average and per-class F1 scores to enable broad comparability across different contexts. Combining data up to four years after forest loss leads to improved model performance overall (macro-average F1 score) and for nearly all individual classes (per-class F1 scores). The classification of degradation drivers and slow-growing plantation benefitted most by incorporating time series data. However, when comparing approaches using only a single image from different years after a forest loss event, images from the first year following an event performed best, both overall (macro-average F1 score) and for most classes (per-class F1 scores), offering a promising strategy for relatively fast analysis of deforestation and degradation drivers following forest loss. We conclude that whilst multi-year imagery is beneficial, relying on a single image from the first year after forest loss still provides valuable and timely insights into the nature of drivers of forest loss.

## 1. Introduction

Commitments such as the European Union Deforestation Law [1] and pledges made at COP26 [2] show international ambition to slow down deforestation in the tropics. However, recent overall trends do not show a decline in forest loss [3]. This is mirrored in Cameroon, which had the 4^th^ highest increase in primary forest loss worldwide in 2022 [4], and the 7^th^ highest tropical primary forest loss in 2023 [3]. Despite this, Cameroon aims to achieve deforestation-free agriculture by 2035 to align with its pledge from the 2021 Glasgow Leaders’ Declaration on Forests and Land Use and the objectives from its 2030 National Development Strategy [5]. Cameroon’s National Adaptation Plan also treats forests as both vulnerable ecosystems and important resources for adaptation, promoting actions like helping communities become more resilient, managing forests sustainably, restoring damaged areas, and reducing carbon emissions through programmes like REDD+ [6]. Its Nationally Determined Contribution (NDC) commitments aim to lower emissions by 32% by 2035 compared to their 2010 baseline [7].

Identifying and tracking deforestation drivers with high spatial and temporal resolution is needed to design and evaluate the potential of interventions [8,9]. Detecting degradation drivers is also necessary since they impact forest functions, properties and services (e.g., carbon storage, biological productivity) [10] and degradation is often the first step before deforestation [11]. Here, we focus on ‘direct’ drivers, meaning the land use leading to the forest loss (e.g., conversion to agriculture land) and not the underlying driver which could have led to the conversion (e.g., change in the price of a commodity). Current solutions to detect these drivers geospatially are either manual [12,13] which can be subjective and time consuming [9]; only cover broad classes with insufficient information about driver types (e.g., ‘plantation’ or ‘small-scale agriculture’ but not crop types) [14–16]; and are often not country-specific [9,17,18]. Some regional studies exist for other countries [11,16,19–25], but as far as we are aware, not for Cameroon (besides our previous own study, [26]. Following an alternative approach focussing on detailed drivers, in [27] we collated an independent dataset of fifteen drivers, and in [26] we describe Cam-Forest, a model to classify these fifteen direct deforestation and degradation drivers for Cameroon.

Examples of direct drivers include smallholder agriculture such as small-scale maize plantation, selective logging, mining, and agro-industrial plantation expansion such as oil palm or rubber. Deforestation drivers can be classified using either single-date or temporal methods, with various methods chosen depending on the use cases, making it challenging to find the most suitable strategy. In addition to our own Cam-ForestNet [26], some studies in other countries used single-date imagery in their classification [16,20–22,25] while others have opted for time series either using composites [11] or temporal segmentation, i.e., partitioning time series into trends or trajectories [23,24]. Various studies in other tropical countries have also used both single-date and temporal classifications separately to compare the two approaches, and tested different methods, including: Convolutional Neural Network (CNN) with the time series concatenated as input, combined with a Long-Short Term Memory module (CNN-LSTM), or with an attention mechanism (CNN-Attention LSTM) [18]; a LSTM, a 3D-CNN with the time dimension treated as a third spatial dimension, a hybrid 2D-CNN/LSTM, a Convolutional Long Short-Term Memory neural network (ConvLSTM), a CNN + Multi-Head Self-Attention model (CNN-MHSA), and a CNN-Transformer [9]; and finally a Temporal Attention U-Net [19].

These comparisons have generally concluded that using temporal data does increase the accuracy of deforestation drivers’ classification, and attention-based models are especially efficient [9,18,19], with improvements attributed to distinct spectral-temporal patterns of crops with different growth patterns [18]. But these studies generally are at large scale and test general driver classifications (e.g., ‘Commercial agriculture’ or ‘Large-scale cropland’). There is reason to believe these findings may not be consistent for detailed drivers in a single country. Indeed, continental scale land-use types may be more easily distinguished by their spatial patterns, while temporal patterns become more useful at the pan-tropical level [9]. Little single-country work (notable exception being [18]) has assessed the potential of temporal methods, and none that we are aware of are in Central Africa or use detailed classes of deforestation drivers. It remains unclear whether a single image is enough, or whether the timing of such an image matters, to produce accurate deforestation driver classification in a context such as Cameroon.

In this paper, we test the value of time series data and temporal features for detailed classification of direct deforestation and degradation drivers in Cameroon with Cam-ForestNet. We hypothesise **(1)** that using images in multiple years will increase the performance of our approach, since visual distinctions between drivers may appear at different times after the forest loss events [18]. Further, we hypothesise **(2)** that, to focus on the deforestation driver and not follow-on land uses, images captured near the time of forest loss event will produce better classifications. This is particularly likely in Cameroon, where land conversions are rapid and newly deforested land often experiences non-linear land use transitions, i.e., multiple transformations in the years following the forest loss [28]. In addition, we consider the suitability of different performance metrics for decision-making and policy in this context.

To test these hypotheses, we here ask two research questions: **(1)** Does combining images from multiple years after the forest loss event improve classification of deforestation and degradation drivers?; **(2)** Does the ‘best’ time (i.e., the timing providing the highest classification performance) after the forest loss event for single image classification differ by driver?

## 2. Materials and methods

### 2.1. Time series dataset construction

We used our previously created reference dataset for classifying deforestation drivers in Cameroon using satellite imagery and auxiliary data, described in [27], as a basis for analysis. There, we selected the single image with the lowest cloud cover in the five years following a known forest loss event using the intersection of Global Forest Change (GFC) with open-access databases with known land uses (see Fig 1 in [27]). Among the data sources, 60% use direct field observations. Data for categories such as ‘Other’, ‘Grassland/Shrubland’, ‘Small-scale oil palm plantation’, and certain points under ‘Other small-scale plantation’ and ‘Wildfire’ are derived from classification algorithms. However, as detailed in the ‘Technical Validation’ section of [27], both the original data creators and our team conducted thorough validation checks to ensure the accuracy and reliability of the dataset.

We used ‘forest loss polygons’ to describe these intersections, which correspond to forest loss areas where we know the follow-up land use, or the direct deforestation/degradation driver. Here, we used a similar approach but, instead of looking at all the images in the five years following the forest loss event, we filter the data to select, for each forest loss polygon, the image with the lowest cloud cover in *each* of the following five years after the loss event (only using images with a cloud cover <20%). This creates a new dataset with up to five images per forest loss event. The 20% cloud cover threshold was chosen based on a process of trial and error, as described in [27]. This threshold provided a practical balance between retaining enough usable images and ensuring adequate visual quality for accurate interpretation.

The GFC product the exact time of the loss is not known, only the year in which the forest loss event occurred. This is because the GFC product identifies forest loss using the maximum annual decline in tree cover and the largest annual decrease in the minimum growing season Normalized Difference Vegetation Index (NDVI) [29]. In our approach, if y represents the year of forest loss, then $y+y_{n}$ indicates the year that occurs $y_{n}$ years after the forest loss event. However, because of the way that GFC is created, this means that the satellite image we use may not have been taken exactly $y_{n}\;$years after the event.

Due to cloud filtering, not all forest loss polygons in [27] provide us with an image each year. Table 1 gives the number of locations (or forest loss polygons) where we have at least one image for each year considered. For instance, there are 2,396 locations where we get at least one image with a cloud cover below 20% from both the first and second year following the forest loss event.

**Table 1:** Number of locations with at least one image with a cloud cover below 20% in each year after the forest loss event. Due to the drop-off in number of locations for all five years, in this analysis we only considered images up to four years after loss.

Years after the forest loss event (e.g., Y1 = first year after the forest loss event)	Number of locations where we have at least one image for all years considered
Y1	2,809
Y1 & Y2	2,396
Y1 & Y2 & Y3	2,207
Y1 & Y2 & Y3 & Y4	2,090
*Y1 & Y2 & Y3 & Y4 & Y5*	*1,215*

**Fig 1:**
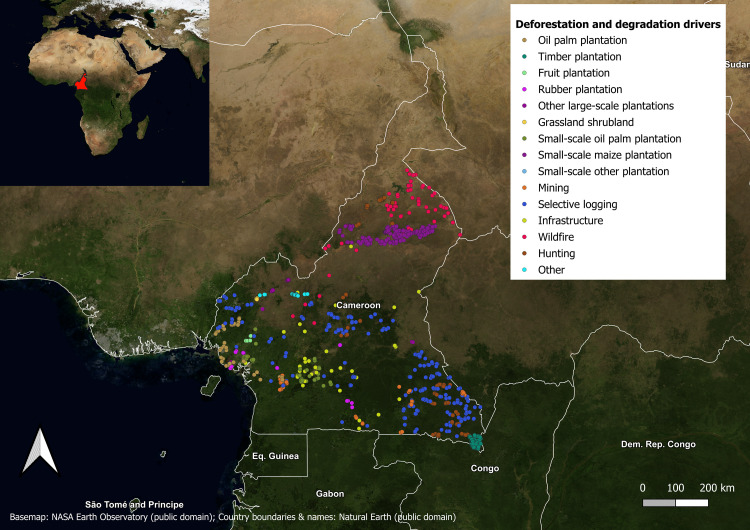
Geographical distribution of the 1,783 locations in the subset used in this study, by class. The study area is Cameroon.

To be able to assess the benefit of multiple years of images, we create a new dataset (representing a subset of [27]) retaining only locations where we have images for each of the four years following the forest loss. We chose this cut-off due to the large drop in number of qualifying locations when looking at five versus four consecutive years (Table 1). We split our data into five cross-validation folds (see Section 2.2 for details) and performed additional filtering to ensure the model is exposed only to spatially disjoint pixels. We excluded locations where forest loss polygons overlap, and filtered the data to maintain a minimum distance of 100 metres between the edges of forest loss polygons in different folds. After these exclusions, a total of 1,783 locations remained, with more than 70% of the data being at least 250 metres from the edges of forest loss polygons in other folds. Fig 1 shows the study area and the geographical distribution of our data.

**Fig 2:**
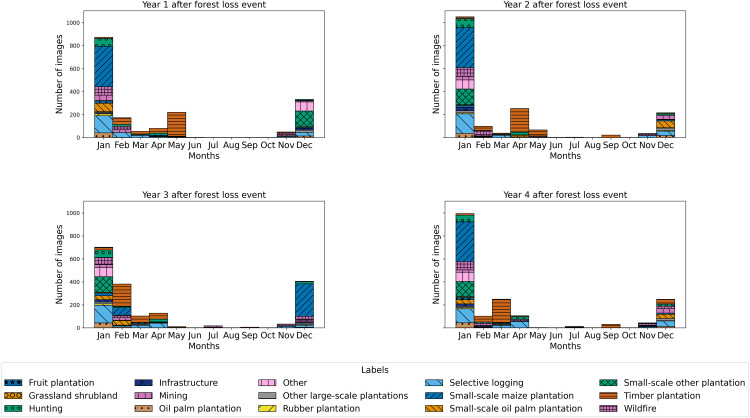
Time distribution of images in the new dataset by class and by time difference (in years) between the image capture and the forest loss event. S2 Table details the number of images per month and dry/rainy season by years after the forest loss event, both overall and by driver class.

Our subset includes around 70% of the original 2,529 locations [26], but with uneven loss of data in the fifteen classes (Table 2). Further, slight differences in the filtering used to create both datasets results in more images for some classes in this dataset compared to the original one. The overwhelming majority of images are from December-February, corresponding to the dry season in Cameroon and therefore the least cloud cover (Fig 2).

**Table 2:** Composition of the dataset used in this study, representing a time series of images created based on the approach in [27], here termed the ‘original dataset’. In bold we highlighted the classes with a small number of images (<50) in our new subset with an asterisk, which we will need to consider when interpreting results. Metrics are based on very small sample sizes, so should be interpreted with caution. Note that it is possible to have more images in the new dataset. In the original dataset [26,27], for each image we 1) selected the image with the lowest cloud cover from the five years following loss and then, 2) discarded images smaller than 10kB, which were blank/partially blank, without replacement. So, if the lowest cloud cover image was in the fifth year, this loss location could be removed from the original dataset, whereas in the new approach it could be retained. In addition, in [26], some images were discarded when splitting the data into training, validation and testing datasets to guarantee a minimum distance of 100 metres between the edges of forest loss polygons in different splits to minimise the impact of spatial autocorrelation.

Class	Number of locations in the original single-image approach [26]	Number of locations in the new subset for time series analyses	% of original locations retained
Oil palm plantation	157	56	36%
Timber plantation	303	346	114%
Fruit plantation (e.g., banana)*	36	29*	81%
Rubber plantation*	109	21*	19%
Other large-scale plantation (e.g., tea, sugarcane)*	98	37*	38%
Grassland/Shrubland*	80	7*	9%
Small-scale oil palm plantation	252	73	29%
Small-scale maize plantation	326	349	107%
Other small-scale plantation	142	140	99%
Mining	166	89	54%
Selective logging	513	262	51%
Infrastructure*	54	38*	70%
Wildfire	118	131	111%
Hunting	117	126	108%
Other	58	79	136%

### 2.2. Five-fold data split for cross validation

We perform a stratified five-fold cross validation to avoid obtaining a potential ‘lucky split’, i.e., an unusually good model performance just because of how the data happens to be split [30]. This is especially prone to happen with a small dataset such as ours where the split could be unrepresentative [30,31]. In five-fold cross-validation, the data is divided into five equally sized parts (or folds). In each round, one of these folds is used for testing, while the remaining four folds are used for training and validating the model. This process is repeated five times, with each fold being used as a test set exactly once. With this approach, we ensure that the model is not simply overfitting to a specific split of the data and can be generalised on new unseen data. While a larger holdout set (e.g., 30%) may further strengthen confidence in generalisability, our combined use of 80:20 splitting and cross-validation offers a reliable compromise between data sufficiency and evaluation rigor.

We apply stratification to preserve class distribution across all folds. Following [26], we did not use a spatially partitioned dataset, as doing so would limit the inclusion of samples from the full diversity of ecological sub-biomes across Cameroon. Instead, to minimise spatial autocorrelation [32–34], we enforced a minimum distance of 100 metres between the edges of forest loss polygons in different folds. This was achieved by starting with a 1 km separation threshold and iteratively relaxing it in 100-metre increments until a viable split was obtained, discarding too-close polygons which could not be assigned. The 100-metre threshold was selected as a pragmatic compromise: it is large enough to reduce local spatial autocorrelation and prevent very close observations from being assigned to different folds, yet not so large that it substantially limits the number of available samples [26]. This threshold has been used in similar fine-scale remote-sensing studies [35,36] precisely because it balances spatial independence with data retention. Within the 80% subset used for training and validation, we further partitioned the data into 80% for training and 20% for validation, again maintaining class balance via stratified sampling. To ensure spatial independence, validation forest loss polygons were required to be at least 100 metres away from any training forest loss polygon. If a validation forest loss polygon violated this condition, it was swapped with a spatially distant training polygon of the same class. In cases where no suitable swap could be identified, the polygon was reassigned to the training set. S1 Table shows the composition of the folds.

### 2.3.Testing hypothesis 1: ‘More data = a more accurate classification?’

We test whether adding more data from different years following the forest loss event will improve the performance of driver classification using our model Cam-ForestNet [26], up to four years after the loss event. We adapt Cam-ForestNet to classify time-series data using the highest class logit. Each image is classified individually and for each image, a logit is calculated for each class (see Fig 2 in (26)]). The final classification for each forest loss location is then based on the year with the highest logit value.

**Fig 3:**
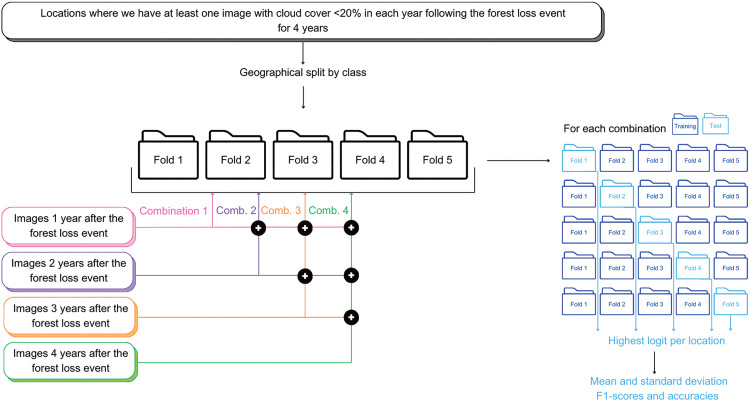
Overview of the methodology used to the test the impact of adding more images from different years after the forest loss event on the performance of Cam-ForestNet.

To test the first hypothesis, we first classify only with the images in the first year after the forest loss event, then consecutively add in images from each year up to four years after the forest loss event. For each combination of years, we maintain the same locations in each fold and include images from additional years. Fig 3 summarises the approach chosen.

We also analyse the mean confusion matrices computed across folds to better understand misclassifications. We specifically examine how misclassifications change when adding data from different years by using a “change matrix.” This matrix is created by subtracting the normalised mean confusion matrix from images taken one year after the forest loss event (Y1) from the normalised mean confusion matrix generated using images from all four consecutive years following the forest loss event (Y1&Y2&Y3&Y4). This helps us identify which misclassifications increase or decrease when combining data from multiple years after the forest loss event.

### 2.4. Testing hypothesis 2: ‘The sooner the better?’

**Fig 4:**
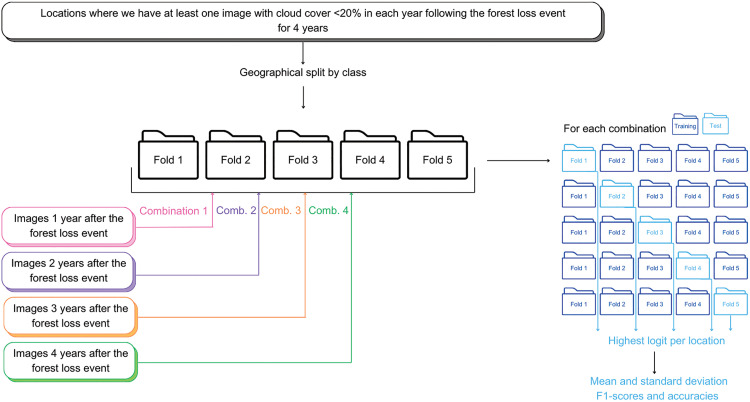
Overview of the methodology used to the test the impact of the time difference between the forest loss event and when the image is taken on the performance of Cam-ForestNet.

We want to test whether there is an ideal time to look at images following the forest loss event, and to study the differences between classes. To do this, we use a very similar approach to Section 2.3., but with classification based on each year’s images individually. We perform the five-fold cross validation for the images in the first year after the forest loss event, then for the second year after the forest loss, and so on. Fig 4 depicts this approach.

To determine whether changes in misclassifications over time may indicate shifts in land use, we analyse the mean confusion matrices obtained across folds. A “change matrix” is created by subtracting the normalised mean confusion matrix from images taken one year after the forest loss event (Y1) from the matrix generated using images taken four years after the event (Y4). For a more detailed understanding of variations between years, we also look at the other change matrices created by subtracting the normalised mean confusion matrix from images taken one year after the forest loss event (Y1) from the matrix generated using images taken three years after the event (Y3), and the matrix generated using images taken two years after the event (Y2).

### 2.5. Comparing multi-year and single-year classification

We also assess whether the temporal patterns exploited by Cam-ForestNet correspond with the ‘ideal timings’ identified with single-year classification models. We examine the distribution of the years, relative to forest loss events, in which the highest logit occurs. Specifically, we count the number of cases where the highest logit, which determines the final classification, corresponds to the first, second, third, or fourth year following the forest loss.

### 2.6. Choice of classification assessment metric

Typical performance metrics used for deep learning classification of land use include recall, precision and F1 score [9,16,17,19,21], and results can vary depending on metric choice. Considering true positives (TP), true negatives (TN), false positives (FP), false negatives (FN), these metrics are defined as [9]:


$$Recall=\frac{correctly\;classified\;positives}{all\;positives}=\frac{TP}{TP+\;FN}$$ (1)

$$Precision=\frac{correctly\;classified\;positives}{all\;classified\;as\;positive}=\frac{TP}{TP+FP}$$ (2)

$$\text{F}\text{1}\;score=\text{2}\frac{Precision*Recall}{Precision+Recall}=\frac{\text{2}TP}{\text{2}TP+FP+FN}$$ (3)

In remote sensing applications, recall is also referred to as producer’s accuracy, while precision is equivalent to user’s accuracy [16,37–39].

These metrics can be derived for each class separately or for the whole dataset [16,21]. Overall (e.g., of the dataset as a whole), precision and recall need to be interpreted with some reservation when there is imbalance between classes [40], which is common in environmental applications. Instead, in that case, it is best to use macro averages, which treat all classes equally regardless of data size [9,17,41].

There have been discussions about how to choose and report performance metrics in the medical field depending on use cases [e.g., 42,43], but this debate is less mature in environmental contexts. In order to select the most relevant metric for comparing results, it is essential to understand how any results will be interpreted and implemented in real-world scenarios [44]. Existing studies classifying deforestation drivers (such as those cited in the introduction) tend to report traditional measures of performance - F1 score, recall, precision – in a machine-learning context, without necessarily evaluating or discussing which are more relevant given the nature of the data and specific real-world applications. By ‘real-world applications’, we mean the practical use of the machine-learning models to provide outputs that are relevant and accessible for decision-making at national or local scales. For instance, recall minimises false negatives and precision minimises false positives, while F1 scores balances recall and precision [45], making the model’s objective crucial when choosing the appropriate metric. The costs, risks, and benefits associated with incorrectly classifying or confusing particular classes must be considered [46,47], whilst the lack of transparency and interpretability from deep learning models has been criticised as limiting their use for high stakes decisions [48]. Beyond standard machine learning performance measures, stakeholders’ preferences, the goal of the model and context-specific limitations or trade-offs need to be considered to guide the model toward producing outcomes that are interpretable, actionable and valuable for decision-making [46]. Determining the right performance metrics is therefore crucial to build confidence in a model.

To choose the performance metric to use to test our hypotheses, we need to consider if it is more important to avoid false negatives (recall), have positive predictions be highly accurate (precision), or to consider both as equally important (F1 score). Our model could be used in different contexts. Examples include: detecting illegal activities (e.g., illegal logging); land use planning and monitoring, especially for sustainable agriculture; preventing land encroachment and helping protect land rights; helping supply chain transparency. Limiting false negatives is important to avoid cases of illegal activities going undetected, especially in regions with high biodiversity or high importance for local communities. However, more false positives could be an issue when detecting these illegal activities, resulting in a waste of resources for investigation but also potential risks on already marginalised communities who would face wrongful penalties. So, whilst maximising recall could be favourable for detection and prevention, maximising precision may be better for regulation enforcement. Our model could be used in both scenarios, hence why we decide here to focus on F1 score to balance both. F1 score has also been used as the main metric for model comparison in similar studies [9,16,18,19]. Nevertheless, we recognise that this measure does not consider true negatives and other complementary metrics we are not discussing here could be useful for a more holistic view of model performance (e.g., fairness or ethical considerations) depending on the use case [45].

## 3. Results

### 3.1. Hypothesis 1: ‘More data = a more accurate classification?’

**Fig 5:**
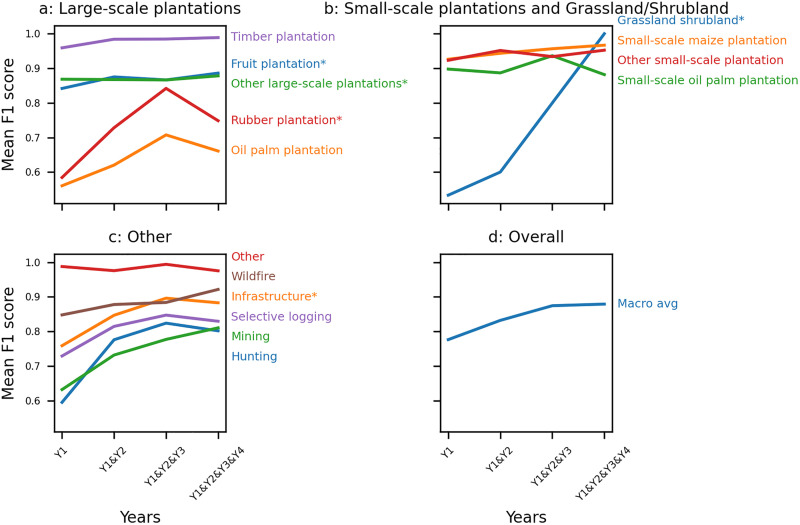
Multi-image model performance for different detailed drivers, categorised by general driver type (a-c) and macro averages of all classes (d). Each panel shows mean F1 scores obtained when taking into account images in the first year (Y1); in the first and second year (Y1&Y2); in the first, second and third year (Y1&Y2&Y3); or in the first, second, third, and fourth years (Y1&Y2&Y3&Y4) following the forest loss event. Asterisks * indicate classes with fewer than 50 images in the dataset (see Table 2).

We trained and tested Cam-ForestNet [26] with images in the first year after the forest loss event, then repeated this consecutively adding in images from each year up to four years after the forest loss event. Fig 5 shows the mean F1 scores obtained across the five folds, by class and overall, for each combination of years. S1 and S2 Figs show the mean recalls and precisions across fold for each combination of years, displaying similar trends as F1 scores.

For the purposes of results interpretation, we define a ‘notable’ change as when the F1 score varies by more than 5%. We categorise each class’s result into four categories: 1) *similar* results no matter the number of years included; 2) *notably better* results by having more years included; 3) *varying* results depending on the years included; and 4) *notably worse* results by adding more years beyond the first year following the forest loss event. We primarily discuss the classes where we consider having ‘enough’ data to draw conclusions (i.e., not identified with an asterisk on Fig 5, meaning there are more than 50 images for the class in the dataset; Table 2), but note that small classes show either *similar* or *notably better* results when including multiple years.

Using the previously defined categories, our results can be summarised as follows. We obtain *similar* results no matter the number of years included for ‘Timber plantation’, ‘Small-scale maize plantation’, ‘Other small-scale plantations’, and ‘Other’. We obtain *notably better* results when we include data from multiple years after the forest loss event for the macro average, ‘Oil palm plantation’, ‘Selective logging’, ‘Hunting’ and ‘Mining’. We obtain *varying* results depending on the years we include for ‘Wildfire’, but which shows a *notably better* F1 score with four years combined compared with only including the first year after the forest loss event. Similarly, we obtain *varying* results for ‘Small-scale oil palm plantation’, which shows a *notably better* F1 score with three years combined and a *notably worse* F1 score when looking across all four years. Finally, we do not obtain *notably worse* results for any class of interest when we include data from multiple years after the forest loss.

We found that standard deviations across folds were generally low for F1 scores compared with recalls and precisions (see S3-S5 Figs). The standard deviation of the macro-average F1 score remains below 5% in all tests. Among the large classes (i.e., > 50 images in the dataset), we obtain standard deviations above 15% only for ‘Oil palm plantation’ (Y1, Y1&Y2).

**Fig 6:**
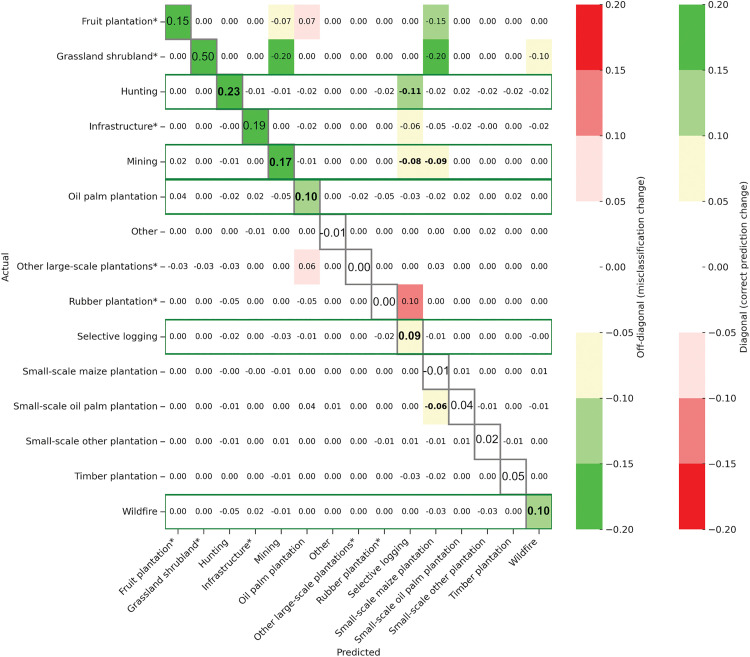
Change matrix created by subtracting the normalised mean confusion matrix from images taken one year after the forest loss event (Y1) from the normalised mean confusion matrix generated using images from all four consecutive years following the forest loss event (Y1&Y2&Y3&Y4). ‘Notable’ changes are shown in colours. Any change higher or lower than 5% is not coloured as we consider it is not a ‘notable’ change. The colours show whether there has been a positive (green) or negative (red) impact on the confusion matrix by adding Y1&Y2&Y3&Y4 in the classification. On the diagonal, emphasised with grey borders and a larger font, a positive number shows an increase in correct classification (green) and a negative number a decrease in correct classification (red). In the rest of the matrix, a positive number shows there has been a higher confusion of two classes (red) while a negative number shows a decrease in the confusion of two classes (green). The green boxes highlight classes where we have an improvement in F1 score when comparing Y1 and Y1&Y2&Y3&Y4. We emphasise the values exhibiting significant variations (i.e., with colours) for classes of interest (i.e., with more than 50 images in the dataset, without an asterisk) in bold.

To better interpret the results above and understand which misclassifications increase or decrease by using images from multiple years after the forest loss event, we generate the change matrix on Fig 6, which shows the difference between the confusion matrices of classification using the first year only and the combination of four consecutive years after the forest loss event (normalised mean confusion matrices for each combination of years are shown in S11-S14 Figs).

Our results show that the effect of including additional images on misclassifications depends on driver types. We therefore split our interpretation of the change matrix in two groups: degradation drivers and all oil palm plantations. Here, we categorise ‘Mining’ as a driver of degradation. In Cameroon, mining mainly occurs through artisanal and small-scale mining (ASM), which primarily leads to forest degradation, though it can also contribute to deforestation [49–51].

#### 3.1.1. Degradation driver classification improves with more data.

Combining images from multiple years had a notable impact on performance for degradation drivers (‘Selective logging’, ‘Wildfire’, ‘Hunting’) (Fig 5). We obtain *notably better* results when we include data from multiple years after the forest loss event for ‘Selective logging’ and ‘Hunting’. Fig 6 shows that ‘Hunting’ is less confused with ‘Selective logging’ when combining images from four years versus the first year. Since they both involve selective rather than clear cutting, we observe a perhaps unsurprising high feature resemblance between ‘Selective logging’ and ‘Hunting’ (S11-S13 Figs). Here, having more data at different times increases the capability of Cam-ForestNet to distinguish between ‘Selective logging’ and ‘Hunting’, especially improving the identification of ‘Hunting’ which is typically harder to detect. We obtain *varying* results for ‘Wildfire’, but with *notably better* results with four years combined compared with using only one year.

Combining images from multiple years improved performance for ‘Mining’, demonstrating the importance of multi-year data for detection (Fig 5). Fig 6 shows that ‘Mining’ is less confused with ‘Selective logging’ and ‘Small-scale maize plantation’ with more years combined. The higher confusion with ‘Selective logging’ in the first year after the forest loss event suggests that mining activities are not necessarily clear cutting and may happen in stages. In Cameroon, most mining is artisanal or small-scale mining, which is consistent with a longer time frame for distinctive features to emerge [49,52,53], which could explain the improved performance seen here. The higher confusion with ‘Small-scale maize plantation’ in the first year after the forest loss event may be explained by co-location of mining and smallholder agriculture, which is a documented phenomenon in the neighbouring Democratic Republic of Congo [25].

#### 3.1.2. Large-scale oil palm plantations need time series data.

Combining images from multiple years after the forest loss event improved performance for ‘Oil palm plantation’, demonstrating the importance of longer-term multi-year data to detect it (Fig 5). Fig 6 shows that ‘Oil palm plantation’ is less confused with ‘Mining’. We suppose it is because oil palm takes a long time to grow, with trees taking at least three years after planting to reach maturity [54,55].

We obtain *varying* results when we include data from multiple years after the forest loss event for ‘Small-scale oil palm plantation’, contrasting with the results for larger industrial areas of oil palm. Nevertheless, Fig 6 and S11-S13 Figs do not identify any class ‘Small-scale oil plantation’ is consistently confused with, though it shows high F1 scores for any year combination (above 0.90).

### 3.2. Hypothesis 2: ‘The sooner the better?’

**Fig 7:**
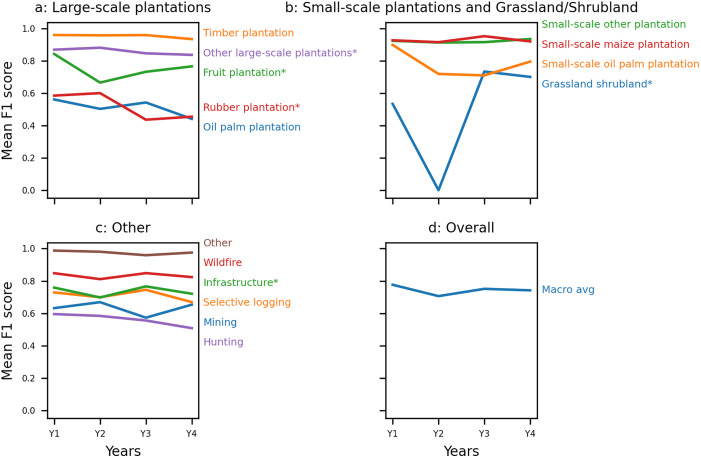
Mean F1 scores obtained when taking into account images in the first (Y1), second (Y2), third (Y3), or fourth year (Y4) following the forest loss event. The asterisk * indicates the classes with fewer than 50 images in the filtered dataset (see Table 2).

We trained and tested Cam-ForestNet [26] with images in each of the first four years after the forest loss event. Fig 7 shows the mean F1 scores obtained across the five folds, by class and overall, for each year following the forest loss event. S6 and S7 Figs show the mean recalls and precisions across folds for each year, which show similar trends as the F1 scores.

As before, we consider there has been a ‘notable’ change when the F1 score varies by more than 5%, and as before we categorise results into four categories: 1) classes where we see no changes between the years; 2) classes where we see *notably better* results when looking close to the forest loss event; 3) classes where close to the forest loss event is when we get results among the best ones but we can see similar results at later times; 4) classes where we get *notably better* results by looking longer after the forest loss event. Here again, we focus interpretation on classes where we consider having ‘enough’ data to draw conclusions (i.e., not identified with an asterisk on Fig 7, meaning there are more than 50 images for the class in the dataset, see Table 2).

For all classes, the best or close to best result was obtained using images taken in the first year after the forest loss event. For some classes, results were *similar* for F1 scores for all years: ‘Timber plantation’, ‘Other small-scale plantation’, ‘Small-scale maize plantation’, ‘Other’, ‘Wildfire’, whilst we obtain *notably better* results in the first year after the forest loss event for ‘Small-scale oil palm plantation’. Some classes had *variable* results, showing similar performance across multiple years without a clear pattern; ‘Oil palm plantation’ and ‘Selective logging’ obtain *notably better* F1 scores in the first and third year following the forest loss event compared to the second and fourth. The macro-average F1 score, ‘Mining’ and ‘Hunting’ shows *similar* results for all years, with slight decreases in the second, third and fourth year, respectively.

As before, we found that standard deviations across folds were generally low for F1 scores compared with recalls and precisions (S8-S10 Figs). The standard deviation of the macro-average F1 score remains below 6% in all tests and among the classes of interest (i.e., with more than 50 images in the dataset), we obtain high standard deviations, i.e., above 15%, only for ‘Oil palm plantation’ (all years).

**Fig 8:**
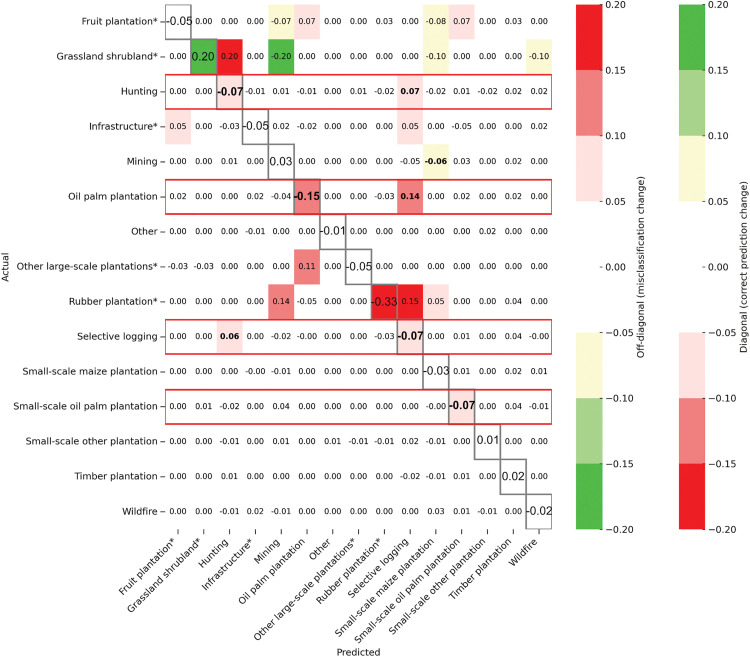
Change matrix created by subtracting the normalised mean confusion matrix from images taken one year after the forest loss event (Y1) from the normalised mean confusion matrix generated using images from the fourth year after the forest loss event (Y4). ‘Notable’ changes are shown in colours. Any change higher or lower than 5% is not coloured as we consider it is not a ‘notable’ change. The colours show whether there has been a positive (green) or negative (red) impact on the confusion matrix by adding Y4 in the classification. On the diagonal, emphasised with grey borders and a larger font, a positive number shows an increase in correct classification (green) and a negative number a decrease in correct classification (red). In the rest of the matrix, a positive number shows there has been a higher confusion of two classes (red) while a negative number shows a decrease in the confusion of two classes (green). The red boxes indicate the classes where we have a decline in F1 score when comparing Y1 and Y4. We emphasise the values exhibiting significant variations (i.e., with colours) for classes of interest (i.e., with more than 50 images in the dataset, without an asterisk) in bold.

To better interpret these results and understand which misclassifications increase or decrease using images at different times, we generate the change matrix, which shows the differences between the first and fourth year after the forest loss event (Fig 8). The normalised mean confusion matrices for each combination of years are on S11 and S15-S17 Figs. Following the methodology outlined in Section 2.5., we compare these results with those from the previous section and examine the ‘best year’ (i.e., with the highest logit) identified by the model for each class in the multi-year classification (see S20-S22 Figs).

We observe that the ‘optimal’ time to analyse imagery varies depending on the type of drivers, and that many of the drivers influenced by the combination of years (Section 3.1) were also affected by the choice of year. As before, we highlight results in two groups: degradation drivers and all oil palm plantations.

#### 3.2.1.Degradation drivers are not easier to distinguish using later imagery alone.

Among degradation drivers, time difference between the forest loss event and image date had an impact on performance for ‘Selective logging’, ‘Hunting’ and ‘Mining’, while ‘Wildfire’ is not impacted (Fig 7). ‘Selective logging’ and ‘Hunting’ show varying performance depending on the year we look at and we obtain *notably* better F1 scores in the first and third year following the forest loss event for ‘Selective logging’, and *notably* worse F1 scores in the fourth year following the forest loss event for ‘Hunting’. Fig 8 and S19 Fig show that variation in performance is linked to how strongly the two classes are confused with one another in different years. Looking at the ‘best year’ (highest logit; S22 Fig), we also see that Year 4 is less commonly chosen for ‘Hunting’.

‘Mining’ obtains similar results for all years, but with a decline in F1 score in the third year after the forest loss event (Fig 7). S18 Fig shows a higher confusion with ‘Small-scale oil palm plantation’ in Year 3 compared with Year 1. We previously found that combining images from multiple years improved performance for ‘Mining’ (Section 3.1.1). Looking at the ‘best year’ (highest logit; S21 and S22 Figs) selected for the multi-year classification, we see that Year 3 is less commonly chosen for ‘Mining’, matching with the drop in performance we see with Year 3 in this section.

#### 3.2.2.Small and large oil palm plantation are both well detected by the first year’s imagery.

‘Oil palm plantation’ shows different performance depending on the years selected, showing the timing of the image impacts the identification of this class (Fig 7). ‘Oil palm plantation’ obtains *notably* better F1 scores in the first and third year following the forest loss event, with more confusion with ‘Selective logging’ and/or ‘Small-scale oil palm plantations’ in other years (Fig 8 and S18 Fig). We previously found that combining images from multiple years improved performance for ‘Oil palm plantation’ (Section 3.1.1). Looking at the ‘best year’ (the highest logit; S21 and S22 Figs) selected for the multi-year classification, we see that Year 2 is less commonly chosen, matching with the drop in performance we see here when looking at single years.

‘Small-scale oil palm plantation’ is the only type of small-scale plantations impacted by the choice of year for images. We obtain *notably* better results in the first year following the forest loss event for this class. We previously found that combining images from multiple years also impacted ‘Small-scale oil palm plantation’ (Section 3.1.1). Looking at the ‘best year’ (highest logit; S20–S22 Figs) selected for the multi-year classification, we see that Year 1 is more commonly chosen for ‘Small-scale oil palm plantation’, matching with the results here which show *notably* better results in the first year following the forest loss event for this class.

## 4. Discussion

### 4.1. Understanding misclassifications beyond model errors

Misclassifications described above may be explained by land use dynamics specific to certain drivers. Unlike ‘Small-scale oil palm plantation’, large-scale ‘Oil palm plantation’ showed *notable* improved performance with multi-year classification. These large-scale plantations tend to have a more consistent and predictable progression compared with small-scale plantations, which typically have more irregular development patterns [25,37,55], which may explain the difference in these results.

With single-year classification, ‘Hunting’ might perform worse in Year 4 because its subtle disturbance signals may be fully overgrown by that time and harder to detect, making it more likely to be misclassified as ‘Selective logging’. While we do not have direct long-term studies on the regrowth of hunting roads in Cameroon, we infer from analogous studies on logging roads [56] that abandoned forest access routes are subject to natural vegetation recovery. Since hunting paths are typically narrower and less intensively cleared than logging roads, it also suggests that regrowth may occur even more rapidly. ‘Mining’ might perform well in Year 1 and 2 because it leaves clear visual traces, such as bare soil and irregular clearings [25,57,58], that might be easier for the model to detect soon after forest loss. The better performance in Year 1 for ‘Oil palm plantation’ may be due to the distinct appearance of newly established plantations (e.g., bare soil, regular planting rows, sharp boundaries) [37,59]. The lower performance for ‘Small-scale oil palm plantation’ after Year 1 might be explained by the fact that these sites are transitioning or mixed-use areas in later years, since smallholder plantations such as oil palm frequently undergo land-use changes and intercropping or mixed cropping in Cameroon [60–63]. Overall, this highlights the importance of incorporating contextualised land-use dynamics in modelling, as classification confusion may not necessarily result from model errors.

The interpretations above are hypotheses informed by common land-use patterns observed in the region, rather than confirmed land-use transitions for our specific data.

### 4.2.Overall, combining images in multiple years following the forest loss event is the best choice

While there are differences in best approach for our individual classes, the macro averages of F1 scores improve when we combine data from multiple years after the forest loss event (Fig 5). Including multiple years in training exposes the model to the full range of visual variation the different classes take over time and the logit-based selection during testing allows the most confident year-specific prediction to drive the final classification. Combining multiple years may also improve model performance by increasing training volume, however we did not find this resulted in better performance for all classes (Fig 5).

For the overall classification, our first hypothesis that combining data from multiple years will improve performance was found to be generally correct and there is no class where we obtain *notably worse* results when we include data from multiple years after the forest loss event. We also find convincing evidence for our second hypothesis, that we obtain one of the highest performance close to the forest loss event, since all classes and macro averages get one of their best F1 scores (+/- 5%) using images in the first year after the forest loss event when looking at single-year analyses. However, our highly curated dataset [27] targets *direct* drivers, using only forest loss events with recorded land use in the same year; which may explain the high performance in the year following the forest loss event.

Previous studies have shown improvements using time series for other locations [9,18,19], which is confirmed here for Cameroon at a national scale and for a more detailed classification of drivers (e.g., no detail about crop types in [9] and only ‘Palm Plantation’ in [18]).

### 4.3.Degradation drivers and ‘Oil palm plantation’ benefit the most from the multi-year analysis

Classes with distinct and evolving visual patterns over time, i.e., ‘Selective logging’, ‘Hunting’, ‘Wildfire, ‘Mining’, and ‘Oil palm plantation’, tend to benefit from multi-year training, as the model may learn to generalise across different disturbance stages and environmental contexts. With single-year training, these classes often show varying performance depending on the specific year, likely due to both their temporal dynamics and intra-class visual variability. In such cases, the model can only learn how a class appears at a particular point in time and may struggle when that appearance changes or lacks distinction from other classes.

Previous studies have similarly shown that the performance for these classes is influenced by the timing of the imagery. For example, in Suriname, the Republic of Congo, and the Democratic Republic of Congo, extending the period for analysing satellite imagery from 1 month to 6 months after logging slightly improved the ability to accurately detect selective logging, likely because signs of disturbance became more visible over time [25]. Another study showed that attention-based spatio-temporal models relied on a single image shortly after wildfire events to detect them, rather than multiple images later in the year following the loss [18]. Mining detection has been shown to improve with spatio-temporal models compared to single-image approaches for pan-tropical studies [18]; and when waiting longer after the disturbance in Suriname, the Republic of Congo, and the Democratic Republic of Congo [25]. Immature oil palm has been found to be confused by other classes, and for example could not be identified in the first three years after planting from other immature monoculture classes such as banana and rubber [55].

### 4.4. Implications and significance

Our results highlight a trade-off between timeliness and accuracy in detecting deforestation drivers, depending on the length of the observation window.

We have seen that overall, the best-performing model (macro-average F1 score: 0.88) is obtained when combining four years’ imagery after the forest loss event, with all classes studied having an average F1 score above 0.80 across the folds, except for ‘Oil palm plantation’. However, this approach will not allow for quick detection following forest loss events, but rather serve longer-timescale analyses and planning. This is especially relevant for land-use change monitoring, long-term landscape management, and post hoc reporting for policy.

In the case of single-year analysis, we saw that looking at the year following the forest loss event is the most effective approach (macro-average F1 score: 0.78). This result is promising because it would help monitor drivers of active deforestation. Knowing the driver behind a deforestation event relatively quickly could help determine whether a ground intervention such as deploying forest rangers or anti-logging patrols would be useful and so help use resources more efficiently. This makes single-year approaches more appropriate for near-real-time monitoring or enforcement-focused applications such as detecting illegal mining, logging, or agricultural encroachment. This also simplifies processing methodology and reduces the resources needed for memory, image processing, and model training time compared to time series classification. Depending on the goal, the most adapted approach could vary between single-year and multi-year methodology.

Our results also raise questions about the most useful way to define the ‘direct driver’, which is particularly challenging when we see dynamics such as transitions in small-scale plantations [60–63]. What we see immediately after the forest loss does not necessarily correlate with the long-term or even medium-term use of the land. It is therefore important to ask, from a policy point of view, what the purpose of monitoring is. If our single-image model gives us accurate information about the follow-up land use after forest loss, it might still be useful to look at time series to better understand land-use dynamics and the longer-term drivers behind deforestation and forest degradation.

For six classes out of fifteen, having time series data proved to be especially effective. This is notably the case for ‘Selective logging’ (+10% performance with all four years’ imagery compared with only the first), ‘Hunting’ (+20%), ‘Mining’ (+18%) and ‘Oil palm plantation’ (+10%). ‘Selective logging’, ‘Hunting’ and ‘Mining’ reach an F1 score above 0.80 only with the multi-year combination, and ‘Oil palm plantation’, ‘Hunting’, and ‘Mining’ perform particularly poorly with only Year 1 (i.e., F1 score < 0.65). This suggests that satellite-based monitoring in the first year following the forest loss event is unlikely sufficient to accurately detect these drivers, which could be an issue for the timely identification of illegal activities which ‘Mining’ and ‘Hunting’ commonly are in Cameroon [64,65]. ‘Selective logging’, also often associated with illegal activity in Cameroon [66], records an average F1 score below 0.80 when using only Year 1 data, which also may limit its detectability in the context of illegal land use tracking [66]. For these classes, using only images in the first year after the forest loss event, we may find improvements with 1) other data, such as additional auxiliary parameters to detect them (e.g., proximity to water for ‘Mining’ [25], proximity to known oil palm concessions for ‘Oil palm plantation’, wildlife or poaching risk layers for ‘Hunting’, known existing logging roads for ‘Selective logging’); or 2) a confidence score to help assess the performance of the classification. Indeed, it is unlikely that the single-image model would be usable for decision-making otherwise.

### 4.5. Limitations and future research direction

In this study we use a dataset developed using all open data identifiable, and after discussion with many experts and stakeholders [27], and which is the largest open dataset of direct drivers of deforestation in Cameroon that we are aware of. However, our dataset is still relatively small, and some features are likely missing. For example, due to the data sources used, we only capture a specific set of land uses, and we likely miss most smaller-scale, illegal or uncontrolled, and quick land-use changes. For instance, in this study, it is interesting to notice that, as time passes, ‘Small-scale maize plantation’ is not particularly confused with other small-scale plantations. Maize grows quickly and is useful to claim land quickly [67,68], which then gives occupancy rights to the users of the land [69,70]. There is evidence indicating that once a land is used and its de facto rights acquired, smallholder farmers in Cameroon may choose to diversify or switch from maize to more lucrative crops when opportunities arise, often a few years later [63,71,72]. In the time frame we look at and with the dataset we have, we do not notice this tendency, but it could be either because we do not look long enough after the forest loss event, or because the data sources we used for labelling maize show longer-standing maize plantations. In addition, we recognise that data availability is skewed towards the dry season, and specific ecozones due to cloud cover, which introduces biases in ecological and spatial representation and can affect the generalisability of the results. As in other studies of tropical forests using optical imagery (e.g., [73,74]), our use of dry-season imagery reflects a common methodological constraint caused by cloud cover during the rainy season. Here, we do not use a cloud masking algorithm and only focus on high quality imagery with low cloud cover using a 20% threshold. This approach prioritises data quality and interpretability, but we acknowledge that it may introduce seasonal sampling bias, especially impacting rainfed and short-cycle agricultural classes.

There are various methods for time-series classification and whilst here we chose the highest logit approach to classify images and combine different years, this is not the only available approach. We use our original Cam-Forest CNN approach to test whether time series provide improvements, using post-hoc aggregation of single-image classifications and not temporal modelling, but we do not explore other techniques such as attention mechanisms or 3D-CNNs for instance, which have been used in other studies [9,18,19]. Nevertheless, our analysis of performance with single images in different years (Section 3.2.) shows that these approaches most likely would not provide improvements using the same timeframe, as we do not see any class where we get *notably* better results compared with the first year after the forest loss event. In addition, we already achieve high F1 scores across most classes (above 0.80, except for ‘Oil palm plantation’), suggesting that the use of a more complex model may not yield substantial benefits. Our results are shown based on F1 scores to assess performance, which may not always be the most suitable approach depending on the application the model is used for and the needs of the users (though we present results for precision and recall in the appendix and find little qualitative difference).

To improve applicability for real-world applications, future work could focus on generating confidence scores to assign for each forest loss event classified, as deep learning has been criticised for not providing interpretable models with accessible outputs that can be used for decision making [48]. A recent study to classify oil palm provided map outputs showing the probability of oil palm in a given pixel to display the model certainty [75]. Such an output could help the use Cam-ForestNet for decision-making at national or local scales. Further research is needed to find the most suitable way to assess the confidence in the model and have a better understanding of uncertainty. Promising options to generate a confidence score include the classification logits, using a softmax function on the classification logits, looking at multiple top classification logits instead of only the highest one, or other techniques to assess uncertainty such as Monte Carlo dropout or Bayesian Neural Networks.

## 5. Conclusion

Understanding the drivers of deforestation and forest degradation is essential for designing effective responses, whether for enforcement, conservation planning, or sustainable land use governance. This study contributes to that goal by demonstrating how incorporating multi-year Earth observation imagery into the Cam-ForestNet approach enhances the classification of direct deforestation and degradation drivers.

The findings indicate that using imagery from up to four years after a forest loss event significantly improves model performance, with a five-fold cross-validation approach yielding average F1 scores above 0.80 for all classes except ‘Oil palm plantation’. Performance particularly improves for degradation drivers, ‘Mining’, and slow-growing large-scale plantations (i.e., ‘Oil palm plantation’), which exhibit both high temporal dynamics and high intra-class visual variability. However, this approach does not support rapid monitoring of deforestation and forest degradation. When considering single-date models, results from using imagery from the year immediately following forest loss were also promising and produced some of the strongest results across all classes. This suggests that timely detection of deforestation drivers is feasible and can support more immediate monitoring and response efforts. Nevertheless, challenges remain for degradation drivers (‘Hunting’, ‘Selective logging’) and long-maturing drivers (‘Oil palm plantation’, ‘Mining’), which appear to require additional data.

This research highlights the trade-offs between approaches that prioritise rapid detection and those that aim for more comprehensive, long-term analysis. It provides useful guidance on how model design can be aligned with different policy goals, such as forest law enforcement, land use planning, or results-based conservation finance. Beyond performance metrics, we also recognise the importance of building models that are transparent, reliable, and usable in real-world contexts. To that end, our future work will focus on improving model interpretability, incorporating uncertainty measures, and ensuring alignment with operational forest governance needs in Cameroon.

## Supporting information

S1 Table Number of locations in each fold. The values correspond to the single-year analysis. When combining multiple years, the number of images is equal to the number of locations listed here multiplied by the number of years combined.
(XLSX)

S2 Table Number of images per month and per dry/rainy season as a function of years after the forest loss event, both in total and disaggregated by driver class. (XLSX)

S1 Fig Mean recalls obtained when taking into account images in the first year (Y1); in the first and second year (Y1&Y2); in the first, second and third year (Y1&Y2&Y3); or in the first, second, third, and fourth years (Y1&Y2&Y3&Y4) following the forest loss event. The asterisk * indicates the classes with fewer than 50 images in the filtered dataset (see Table 2).
(PNG)

S2 Fig Mean precisions obtained when taking into account images in the first year (Y1); in the first and second year (Y1&Y2); in the first, second and third year (Y1&Y2&Y3); or in the first, second, third, and fourth years (Y1&Y2&Y3&Y4) following the forest loss event. The asterisk * indicates the classes with fewer than 50 images in the filtered dataset (see Table 2).
(PNG)

S3 Fig Means and standard deviations of F1 scores for all folds by class when taking into account images in the first year (Y1); in the first and second year (Y1&Y2); in the first, second and third year (Y1&Y2&Y3); or in the first, second, third, and fourth years (Y1&Y2&Y3&Y4). (PNG)

S4 Fig Means and standard deviations of recalls for all folds by class when taking into account images in the first year (Y1); in the first and second year (Y1&Y2); in the first, second and third year (Y1&Y2&Y3); or in the first, second, third, and fourth years (Y1&Y2&Y3&Y4). (PNG)

S5 Fig Means and standard deviations of precisions for all folds by class when taking into account images in the first year (Y1); in the first and second year (Y1&Y2); in the first, second and third year (Y1&Y2&Y3); or in the first, second, third, and fourth years (Y1&Y2&Y3&Y4). (PNG)

S6 Fig Mean recalls obtained when taking into account images in the first (Y1), second (Y2), third (Y3), or fourth year (Y4) following the forest loss event. The asterisk * indicates the classes with fewer than 50 images in the filtered dataset (see Table 2).
(PNG)

S7 Fig Mean precisions obtained when taking into account images in the first (Y1), second (Y2), third (Y3), or fourth year (Y4) following the forest loss event. The asterisk * indicates the classes with fewer than 50 images in the filtered dataset (see Table 2).
(PNG)

S8 Fig Means and standard deviations of F1 scores for all folds by class when taking into account images in the first (Y1), second (Y2), third (Y3), or fourth year (Y4) following the forest loss event. (PNG)

S9 Fig Means and standard deviations of recalls for all folds by class when taking into account images in the first (Y1), second (Y2), third (Y3), or fourth year (Y4) following the forest loss event. (PNG)

S10 Fig Means and standard deviations of precisions for all folds by class when taking into account images in the first (Y1), second (Y2), third (Y3), or fourth year (Y4) following the forest loss event. (PNG)

S11 Fig Mean confusion matrix for all folds when taking into account images in the first year following the forest loss event. (PNG)

S12 Fig Mean confusion matrix for all folds when taking into account images in the first and second years following the forest loss event. (PNG)

S13 Fig Mean confusion matrix for all folds when taking into account images in the first, second, and third years following the forest loss event. (PNG)

S14 Fig Mean confusion matrix for all folds when taking into account images in the first, second, third, and fourth years following the forest loss event. (PNG)

S15 Fig Mean confusion matrix for all folds when taking into account images in the second year following the forest loss event. (PNG)

S16 Fig Mean confusion matrix for all folds when taking into account images in the third year following the forest loss event. (PNG)

S17 Fig Mean confusion matrix for all folds when taking into account images in the fourth year following the forest loss event. (PNG)

S18 Fig Change matrix created by subtracting the normalised mean confusion matrix from images taken one year after the forest loss event (Y1) from the normalised mean confusion matrix generated using images from the third year after the forest loss event (Y3). The colours show whether there has been a positive (green) or negative (red) impact on the confusion matrix by adding Y3 in the classification. On the diagonal, a positive number shows an increase in correct classification (green) and a negative number a decrease in correct classification (red). In the rest of the matrix, a positive number shows there has been a higher confusion of two classes (red) while a negative number shows a decrease in the confusion of two classes (green). Any changes higher or lower than 5% is in white as we consider it is not a ‘notable’ change.
(PNG)

S19 Fig Change matrix created by subtracting the normalised mean confusion matrix from images taken one year after the forest loss event (Y1) from the normalised mean confusion matrix generated using images from the fourth year after the forest loss event (Y2). The colours show whether there has been a positive (green) or negative (red) impact on the confusion matrix by adding Y2 in the classification. On the diagonal, a positive number shows an increase in correct classification (green) and a negative number a decrease in correct classification (red). In the rest of the matrix, a positive number shows there has been a higher confusion of two classes (red) while a negative number shows a decrease in the confusion of two classes (green). Any changes higher or lower than 5% is in white as we consider it is not a ‘notable’ change.
(PNG)

S20 Fig Years with the highest logit chosen in the test step when taking into account images in the first (Y1) and second (Y2) years following the forest loss event. (PNG)

S21 Fig Years with the highest logit chosen in the test step when taking into account images in the first (Y1), second (Y2), and third (Y3) years following the forest loss event. (PNG)

S22 Fig Years with the highest logit chosen in the test step when taking into account images in the first (Y1), second (Y2), third (Y3), and fourth (Y4) years following the forest loss event. (PNG)
