# Supplementary figures and images for: The fast and the focused: Balancing timely and accurate classification of deforestation and degradation drivers using remote sensing
Source: PLoS One. 2026 Feb 6;21(2):e0340610. doi: 10.1371/journal.pone.0340610 (PMC12880707; doi:10.1371/journal.pone.0340610)

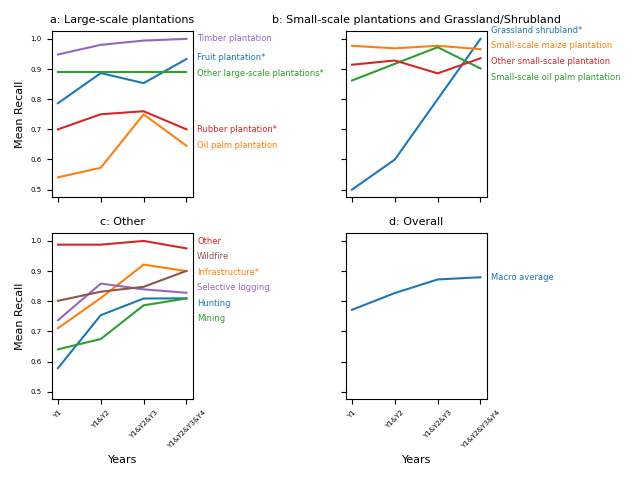

Supplement: S1 Fig — The asterisk * indicates the classes with fewer than 50 images in the filtered dataset (see Table 2). (PNG) [file pone.0340610.s003.png]

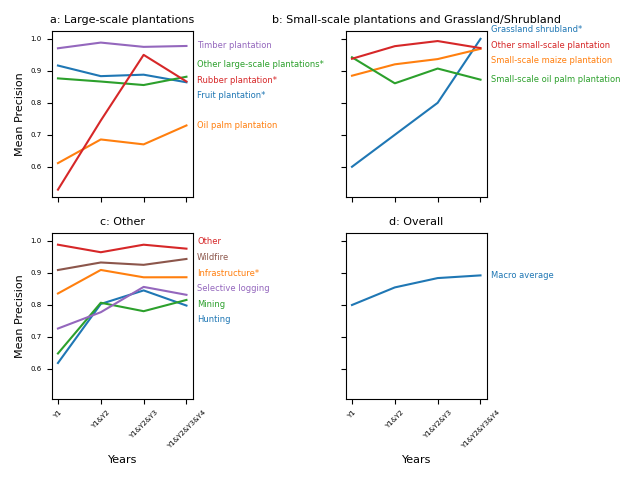

Supplement: S2 Fig — The asterisk * indicates the classes with fewer than 50 images in the filtered dataset (see Table 2). (PNG) [file pone.0340610.s004.png]

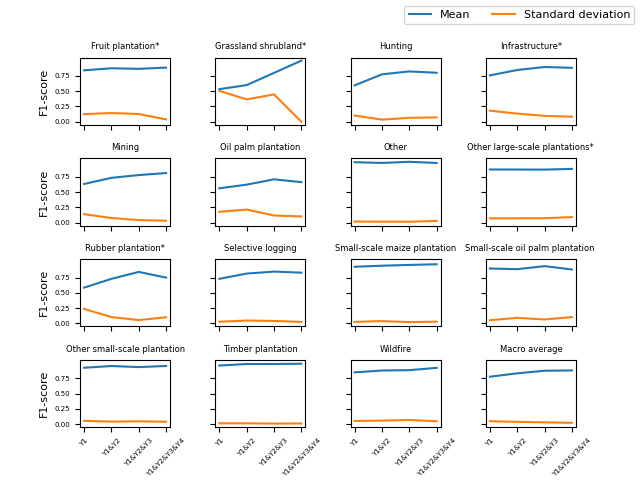

Supplement: S3 Fig — (PNG) [file pone.0340610.s005.png]

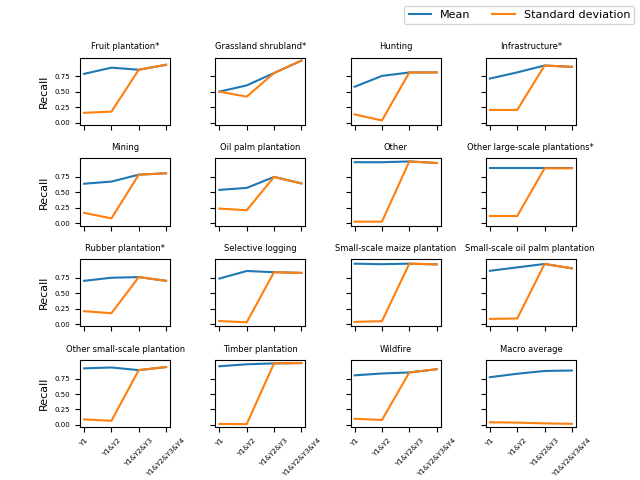

Supplement: S4 Fig — (PNG) [file pone.0340610.s006.png]

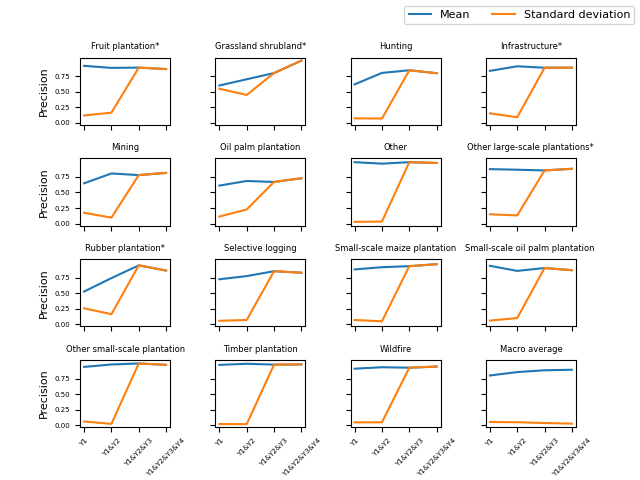

Supplement: S5 Fig — (PNG) [file pone.0340610.s007.png]

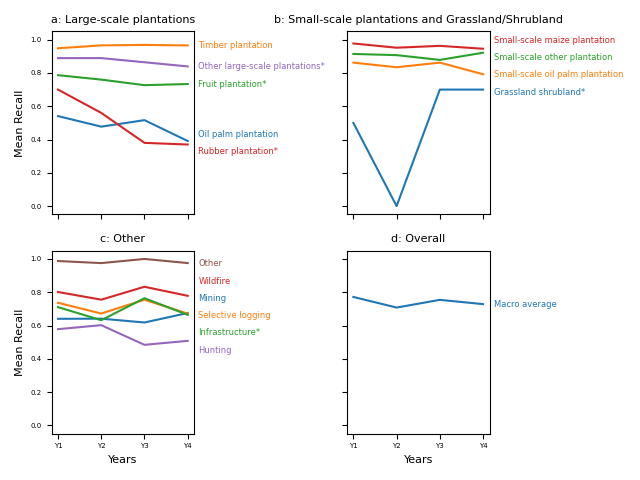

Supplement: S6 Fig — The asterisk * indicates the classes with fewer than 50 images in the filtered dataset (see Table 2). (PNG) [file pone.0340610.s008.png]

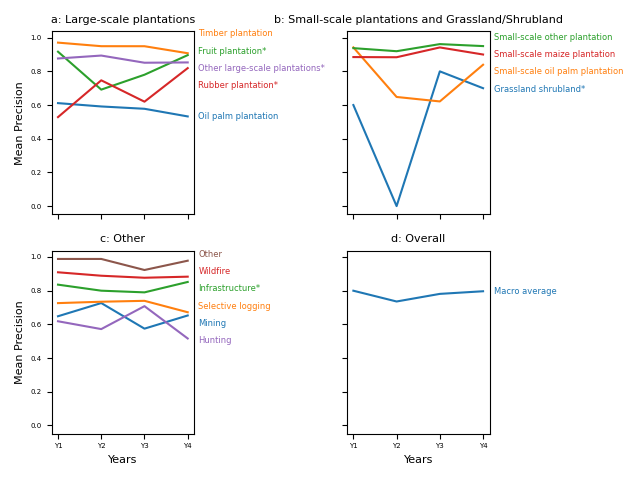

Supplement: S7 Fig — The asterisk * indicates the classes with fewer than 50 images in the filtered dataset (see Table 2). (PNG) [file pone.0340610.s009.png]

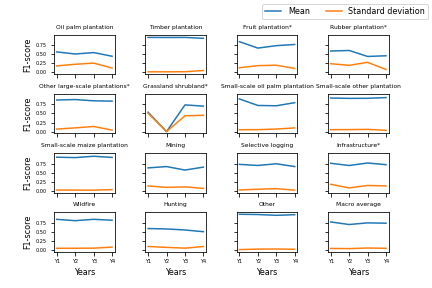

Supplement: S8 Fig — (PNG) [file pone.0340610.s010.png]

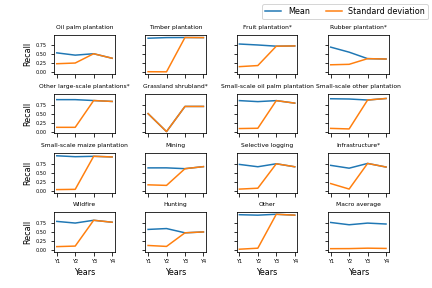

Supplement: S9 Fig — (PNG) [file pone.0340610.s011.png]

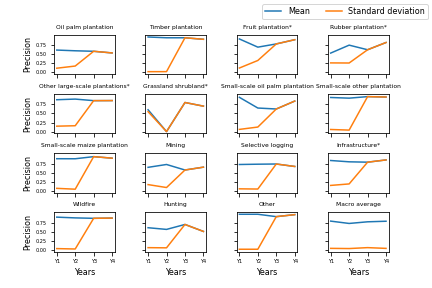

Supplement: S10 Fig — (PNG) [file pone.0340610.s012.png]

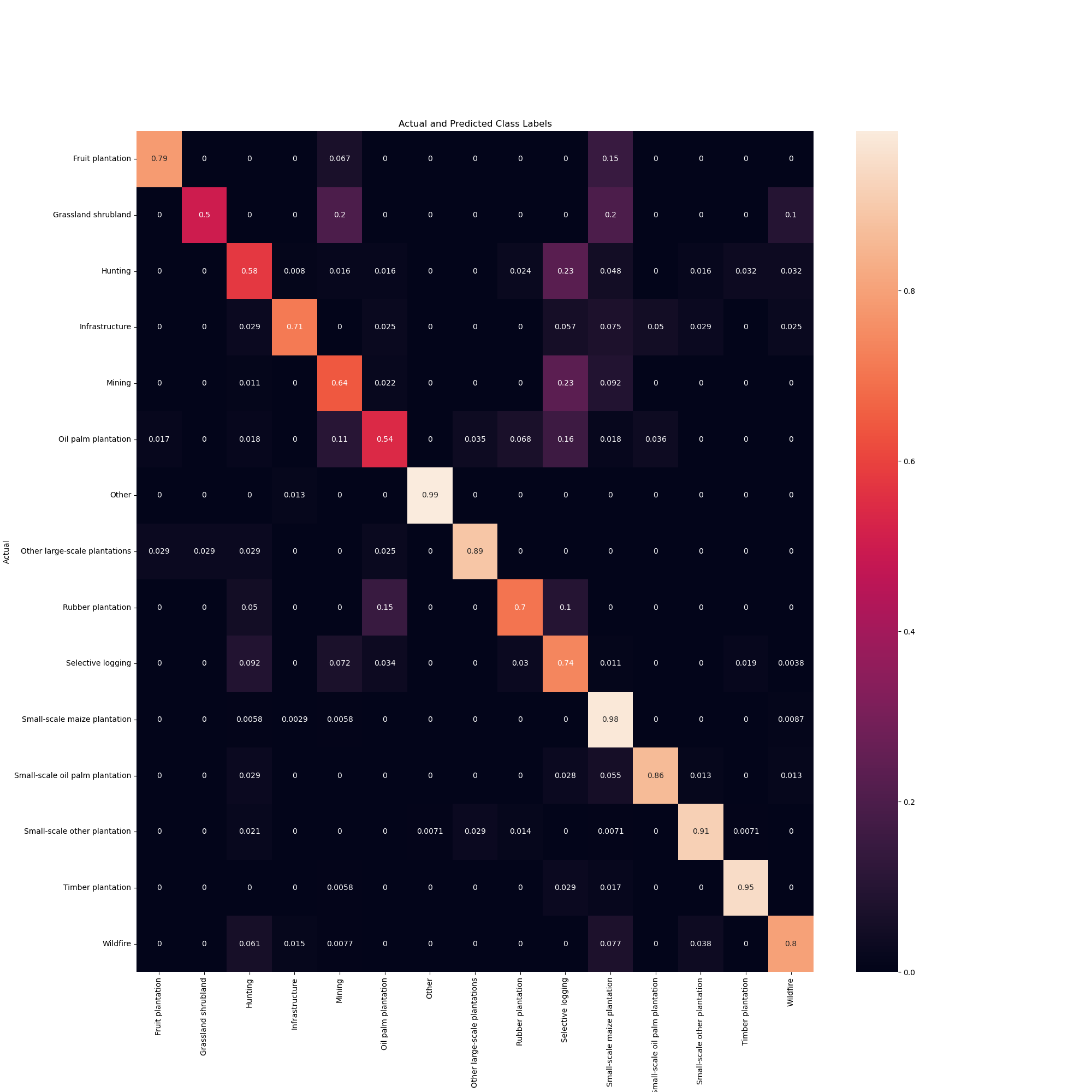

Supplement: S11 Fig — (PNG) [file pone.0340610.s013.png]

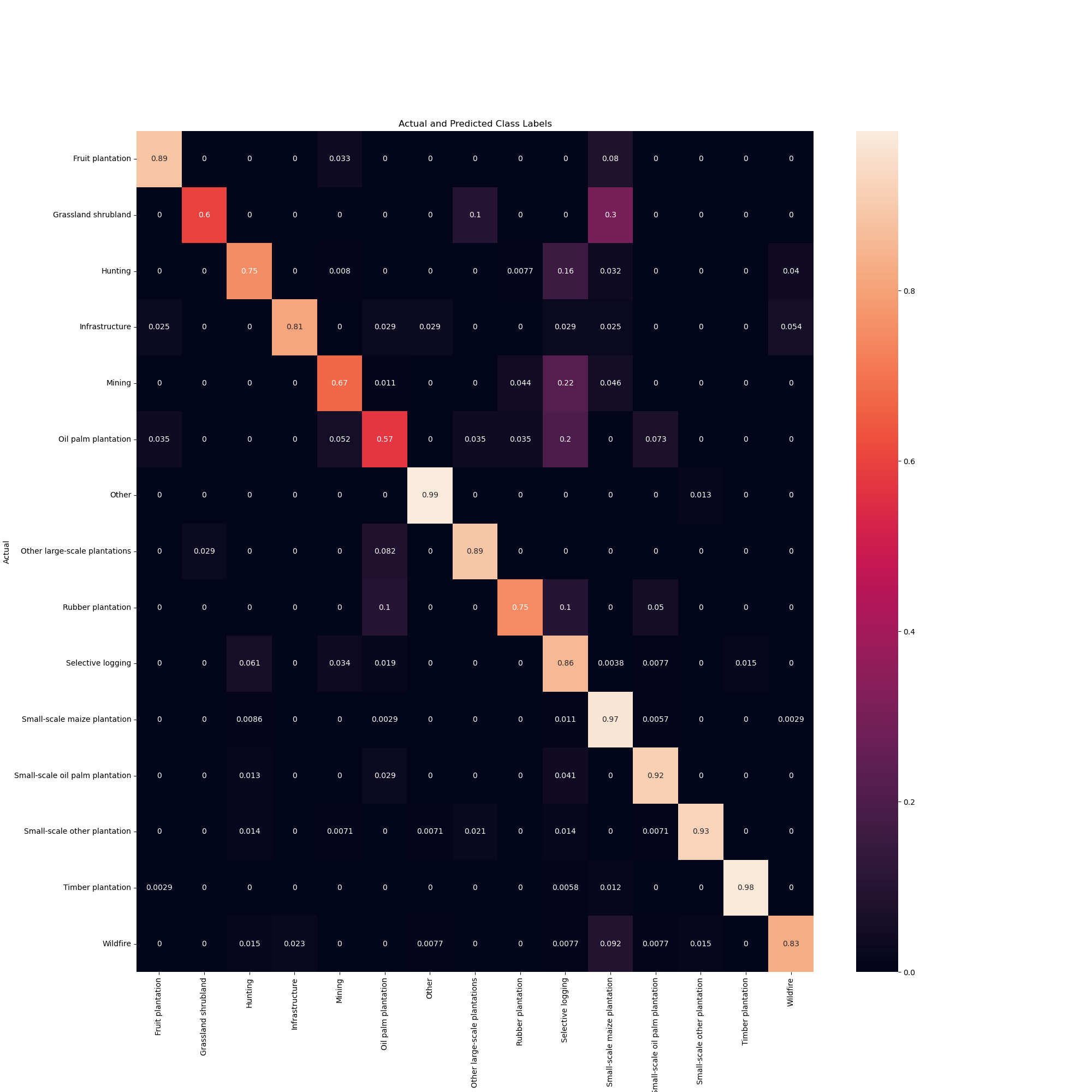

Supplement: S12 Fig — (PNG) [file pone.0340610.s014.png]

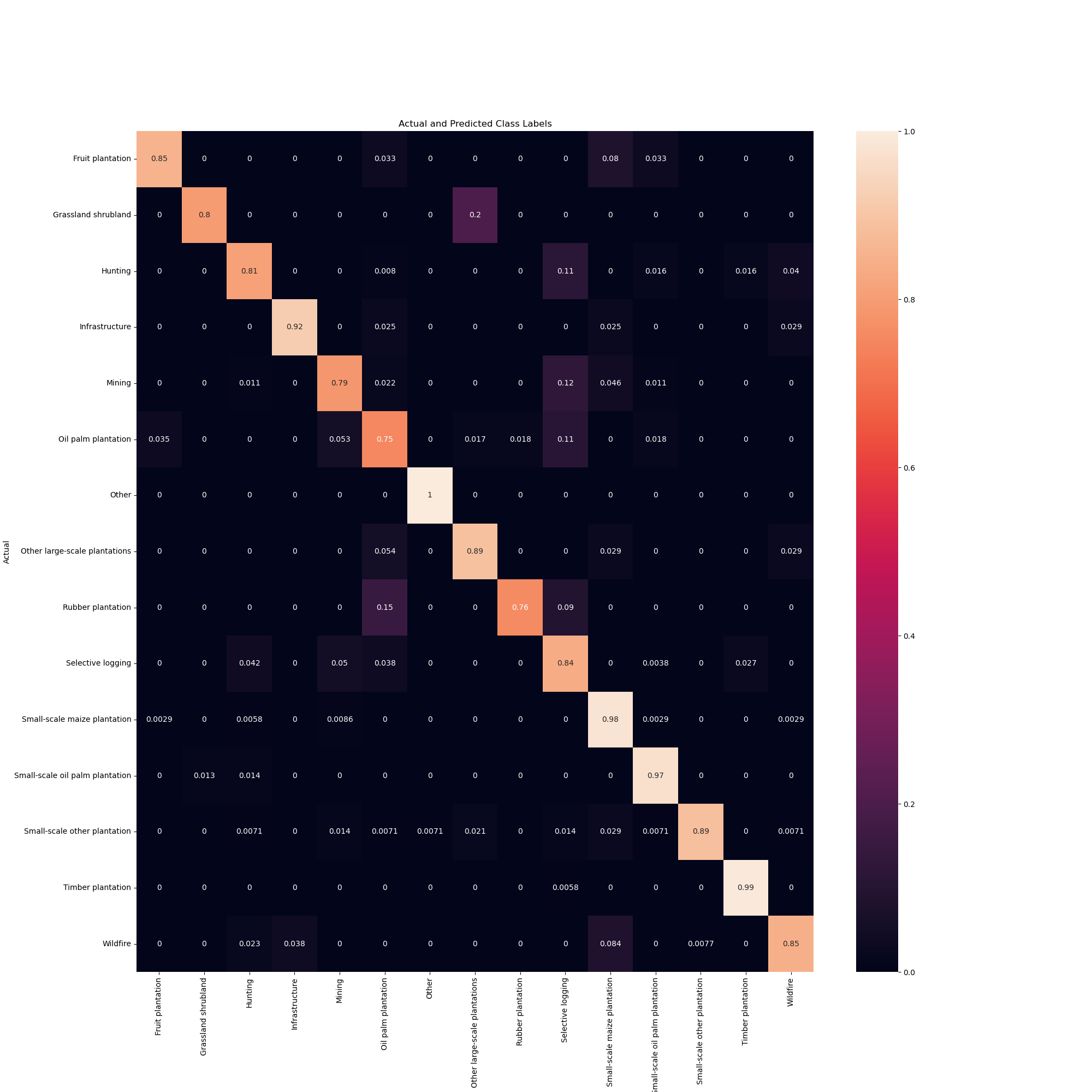

Supplement: S13 Fig — (PNG) [file pone.0340610.s015.png]

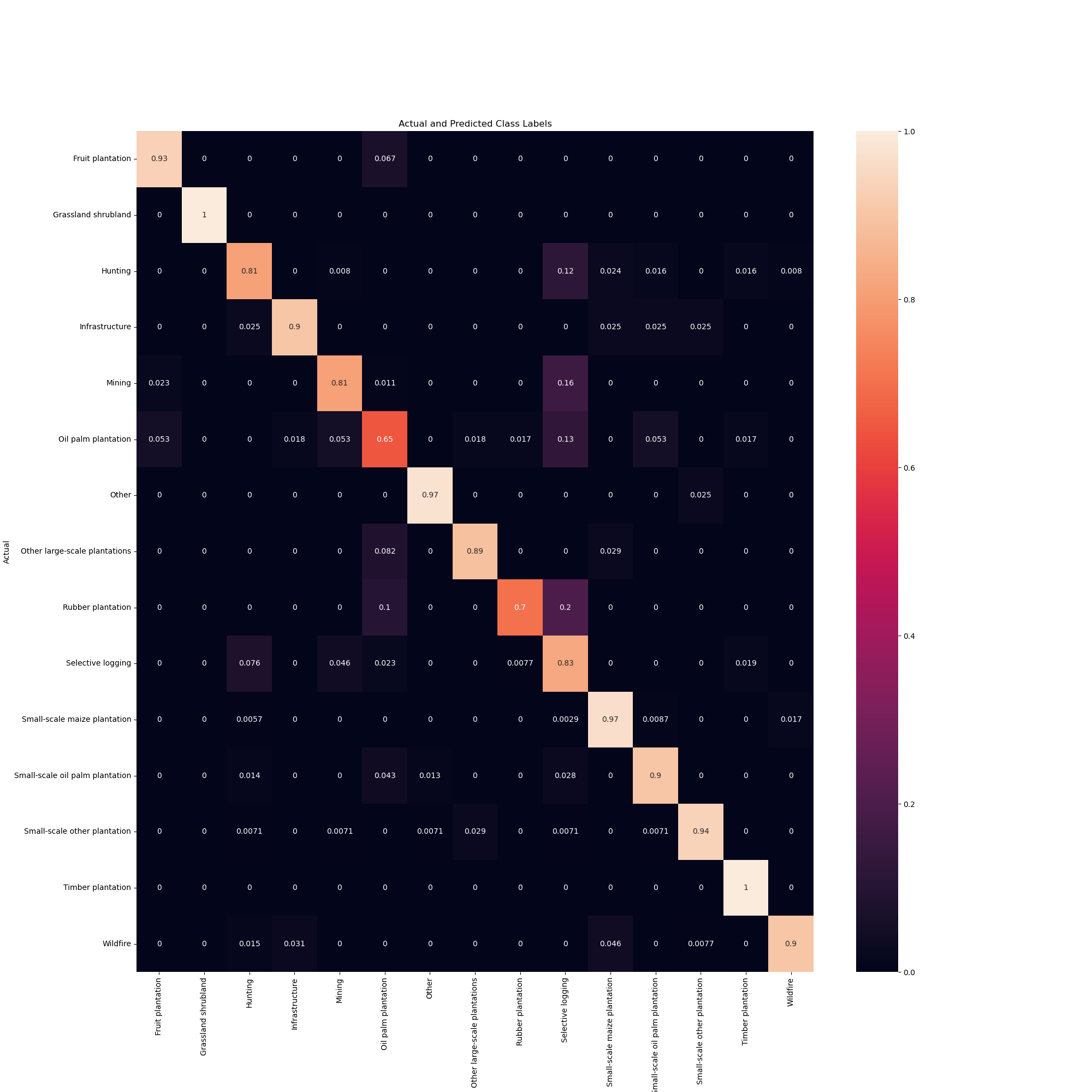

Supplement: S14 Fig — (PNG) [file pone.0340610.s016.png]

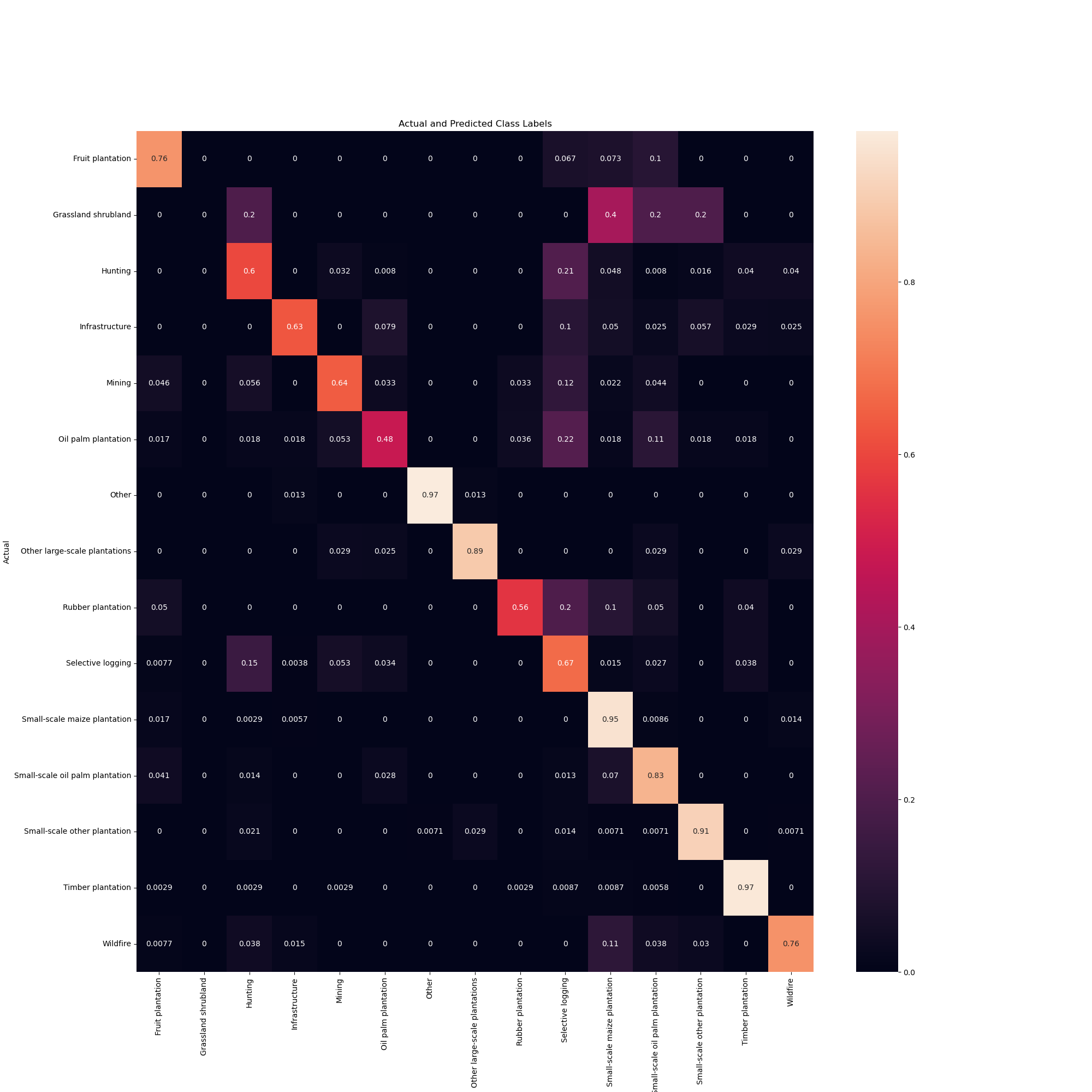

Supplement: S15 Fig — (PNG) [file pone.0340610.s017.png]

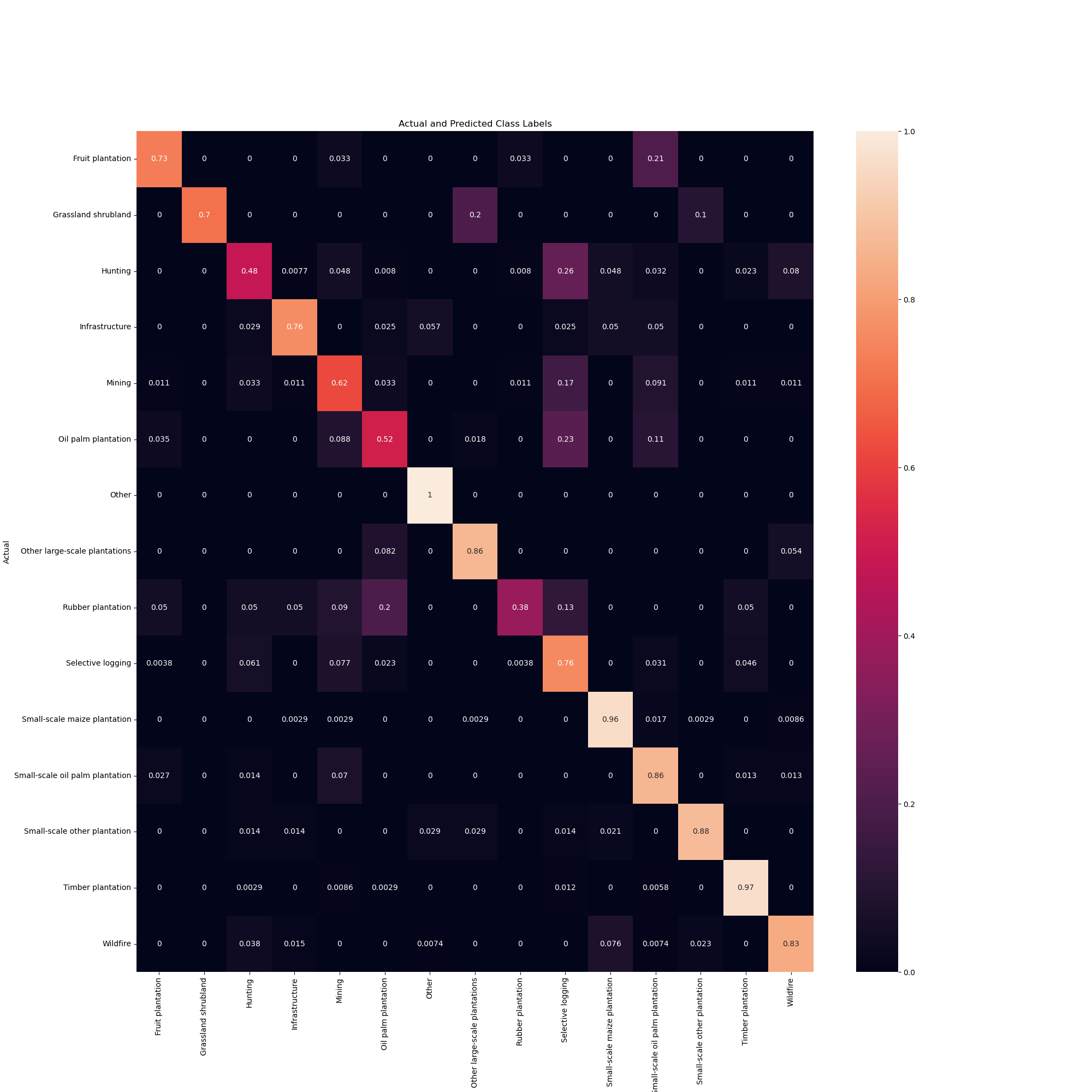

Supplement: S16 Fig — (PNG) [file pone.0340610.s018.png]

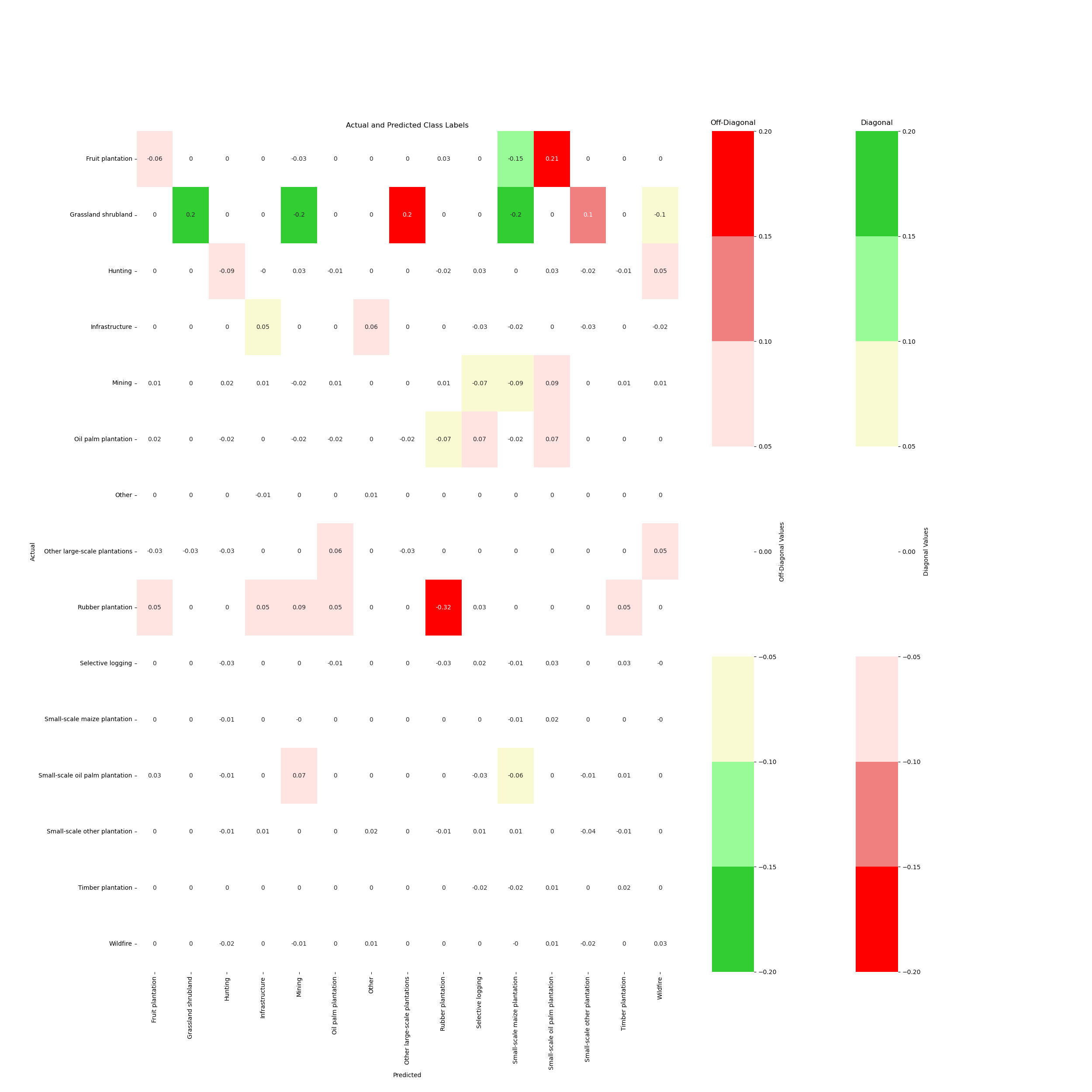

Supplement: S17 Fig — (PNG) [file pone.0340610.s019.png]

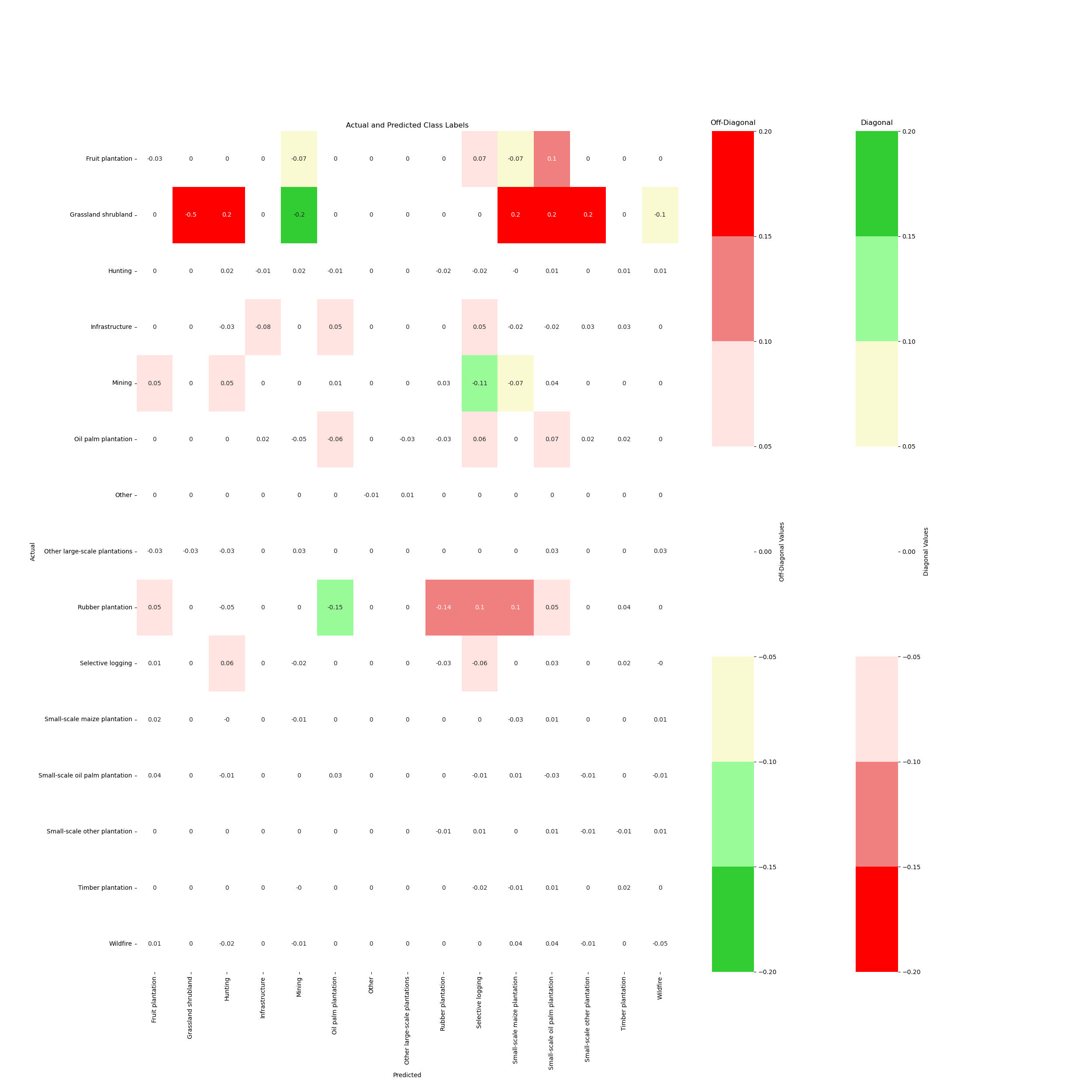

Supplement: S19 Fig — The colours show whether there has been a positive (green) or negative (red) impact on the confusion matrix by adding Y2 in the classification. On the diagonal, a positive number shows an increase in correct classification (green) and a negative number a decrease in correct classification (red). In the rest of the matrix, a positive number shows there has been a higher confusion of two classes (red) while a negative number shows a decrease in the confusion of two classes (green). Any changes higher or lower than 5% is in white as we consider it is not a ‘notable’ change. (PNG) [file pone.0340610.s021.png]

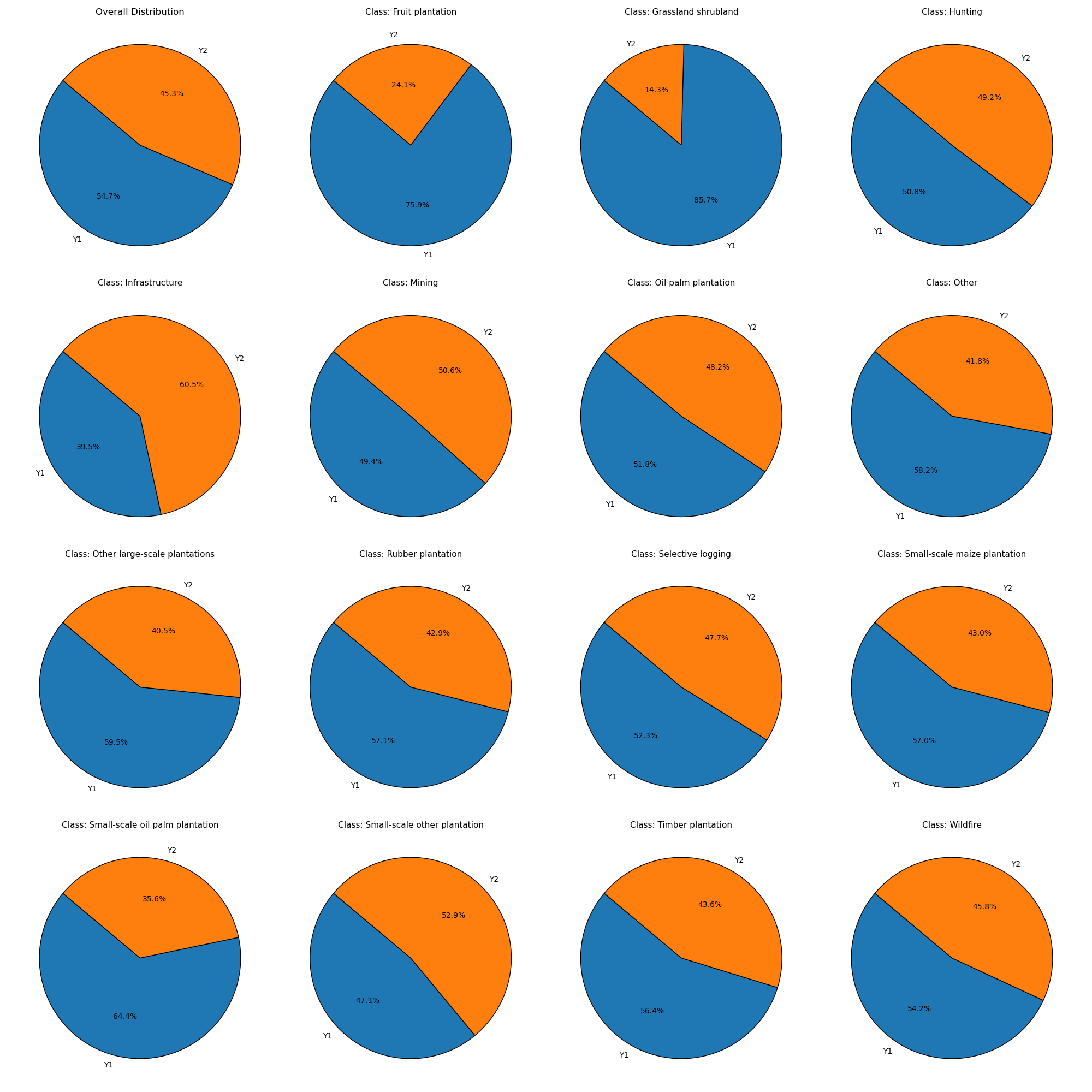

Supplement: S20 Fig — (PNG) [file pone.0340610.s022.png]

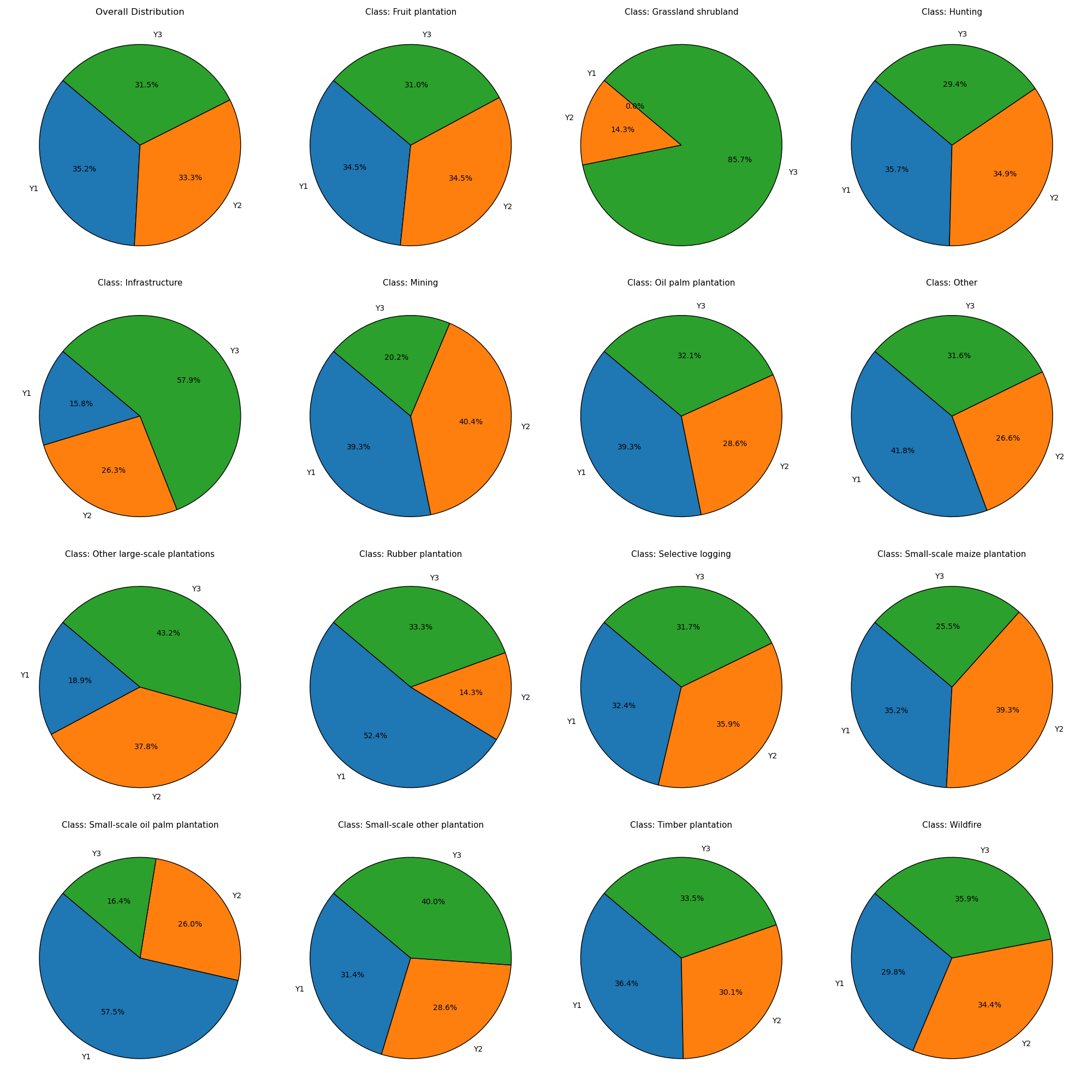

Supplement: S21 Fig — (PNG) [file pone.0340610.s023.png]

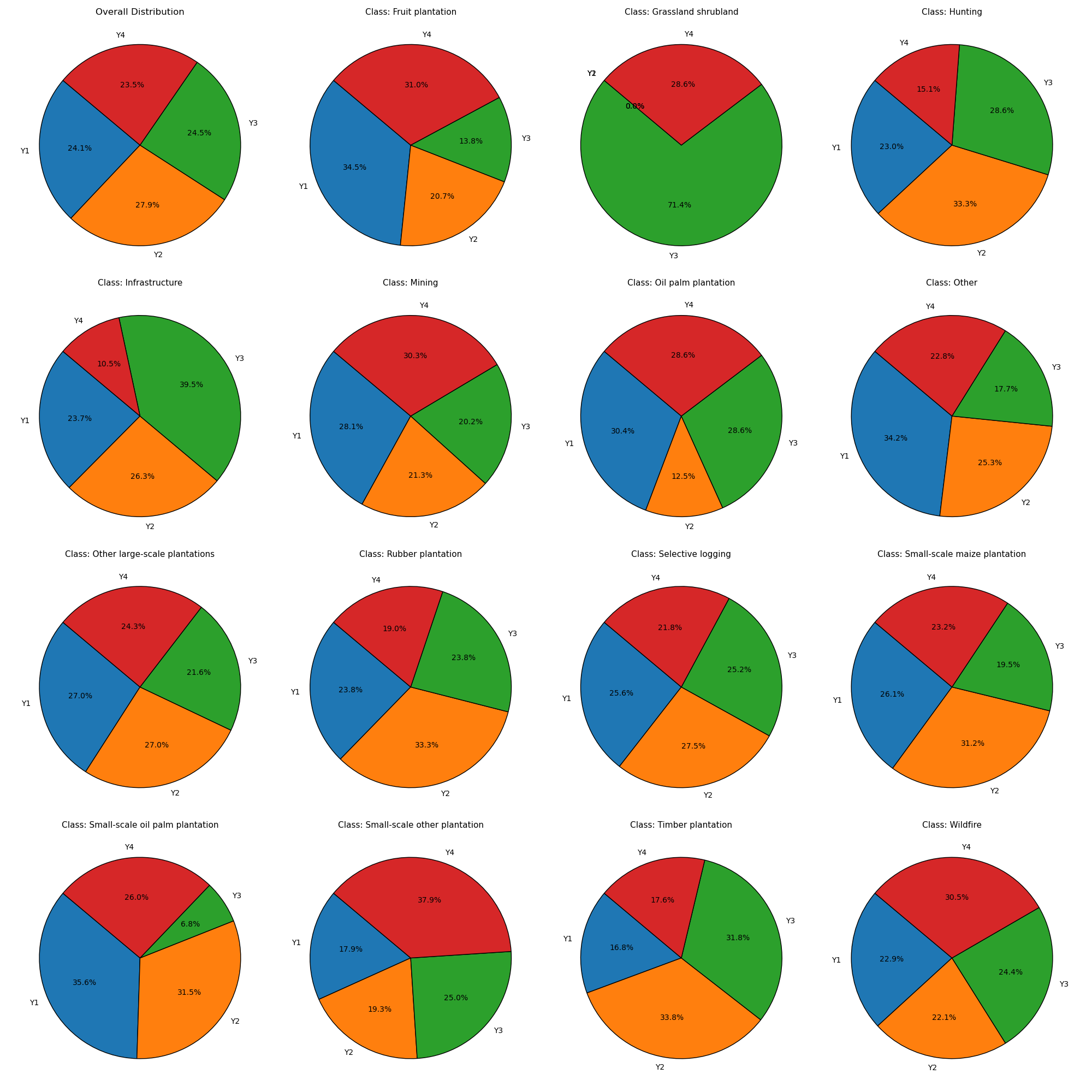

Supplement: S22 Fig — (PNG) [file pone.0340610.s024.png]
